# Supplementary material for: Association between migration and severe maternal outcomes in high-income countries: Systematic review and meta-analysis
Source: PLoS Med. 2023 Jun 22;20(6):e1004257. doi: 10.1371/journal.pmed.1004257 (PMC10328365; doi:10.1371/journal.pmed.1004257)
Supplement: S1 Table — (DOCX) [file pmed.1004257.s001.docx]

S1 Table. Literature search algorithm on Medline via PubMed

| **Set#** | **Search term** |
| --- | --- |
| S1 | ((Migrant[tiab]) OR (Migrants[tiab]) OR (Immigrant[tiab]) OR (Immigrants[tiab]) OR (emigrant[tiab]) OR (emigrants[tiab]) OR (“Foreign-born“[tiab]) OR (“Foreign born“[tiab]) OR (Migration[tiab]) OR (Migrations[tiab]) OR (Immigration[tiab]) OR (Immigrations[tiab]) OR (Emigration and Immigration[mh]) OR (transients and migrants[mh]) OR (“maternal country of birth”[tiab]) OR (“maternal region of birth”[tiab]) OR (“maternal place of birth”[tiab]) OR (“legal status”[tiab]) OR (“administrative status”[tiab]) OR (“asylum seeker”[tiab]) OR (“asylum seekers”[tiab]) OR (refugee[tiab]) OR (refugees[tiab]) OR (refugees[mh]) OR (expatriate[tiab]) OR (expatriates[tiab]) OR (exile[tiab]) OR (exiles[tiab])) |
| S2 | ((maternal mortality[tiab]) OR ("maternal mortalities"[tiab]) OR (maternal mortality[mh]) OR ("maternal death"[tiab]) OR ("maternal deaths"[tiab]) OR (maternal death[mh]) OR ("Pregnancy-related mortality"[tiab]) OR ("Pregnancy-related mortalities"[tiab]) OR ("Pregnancy-associated mortality"[tiab]) OR ("Pregnancy-associated mortalities"[tiab]) OR ("Maternal near-miss"[tiab]) OR ("severe maternal morbidity"[tiab]) OR ("severe maternal morbidities"[tiab]) OR ("severe acute maternal morbidity"[tiab]) OR ("severe acute maternal morbidities"[tiab]) OR ("Obstetric hemorrhage"[tiab]) OR ("Obstetric hemorrhages"[tiab]) OR ("Obstetric haemorrhage"[tiab]) OR ("Obstetric haemorrhages"[tiab]) OR ("Postpartum hemorrhage"[tiab]) OR ("Postpartum hemorrhages"[tiab]) OR ("Postpartum haemorrhage"[tiab]) OR ("Postpartum haemorrhages"[tiab]) OR ("Postpartum hemorrhage"[mh]) OR (“peripartum hysterectomy”[tiab]) OR (“peripartum hysterectomies”[tiab]) OR (“peri partum hysterectomy”[tiab]) OR (“peri partum hysterectomies”[tiab]) OR (“pregnancy-related hysterectomy”[tiab]) OR (Eclampsia[tiab]) OR (Eclampsias[tiab]) OR (Eclampsia[mh]) OR (Preeclampsia[tiab]) OR (Preeclampsias[tiab]) OR (“Pre eclampsia”[tiab]) OR (“Pre eclampsias”[tiab]) OR (“Pregnancy toxemia”[tiab]) OR (“Pregnancy toxemias”[tiab]) OR (“Toxemia of pregnancy”[tiab]) OR (“Toxemia of pregnancies”[tiab]) OR (Preeclampsia[mh]) OR ("Maternal sepsis"[tiab]) OR (“uterine rupture”[tiab]) OR (“uterine ruptures”[tiab]) OR (uterine rupture[mh]) OR (“Maternal admission to the intensive care unit”[tiab]) OR (“Maternal intensive care unit admission”[tiab]) OR (“Maternal admission to an intensive care unit”[tiab])) |
| S3 | ((maternal[tiab]) AND (intensive care units[mh])) |
| S4 | S1 AND (S2 OR S3) |
| S5 | S4 AND ("1990"[Date - Publication] : "2023"[Date - Publication]) |
