# Supplementary material for: Association between migration and severe maternal outcomes in high-income countries: Systematic review and meta-analysis
Source: PLoS Med. 2023 Jun 22;20(6):e1004257. doi: 10.1371/journal.pmed.1004257 (PMC10328365; doi:10.1371/journal.pmed.1004257)
Supplement: S2 Table — (DOCX) [file pmed.1004257.s002.docx]

S2 Table. Literature search algorithm on Embase

| **Set#** | **Search term** |
| --- | --- |
| S1 | (Migrant:ti,ab OR Migrants:ti,ab OR Immigrant:ti,ab OR Immigrants:ti,ab OR emigrant:ti,ab OR emigrants:ti,ab OR ‘Foreign-born’:ti,ab OR ‘Foreign born’:ti,ab OR Migration:ti,ab OR Migrations:ti,ab OR Immigration:ti,ab OR Immigrations:ti,ab OR ‘maternal country of birth’:ti,ab OR ‘maternal region of birth’:ti,ab OR ‘maternal place of birth’:ti,ab OR ‘legal status’:ti,ab OR ‘administrative status’:ti,ab OR ‘asylum seeker’:ti,ab OR ‘asylum seekers’:ti,ab OR refugee:ti,ab OR refugees:ti,ab OR expatriate:ti,ab OR expatriates:ti,ab OR exile:ti,ab OR exiles:ti,ab OR 'migrant'/exp OR 'immigration'/exp OR 'legal status'/exp) |
| S2 | (‘maternal mortality’:ti,ab OR ‘maternal mortalities’:ti,ab OR ‘maternal death’:ti,ab OR ‘maternal deaths’:ti,ab OR ‘Pregnancy-related mortality’:ti,ab OR ‘Pregnancy-related mortalities’:ti,ab OR ‘Pregnancy-associated mortality’:ti,ab OR ‘Pregnancy-associated mortalities’:ti,ab OR 'maternal mortality'/exp OR 'maternal death'/exp OR ‘Maternal near-miss’:ti,ab OR 'maternal near miss'/exp OR ‘severe maternal morbidity’:ti,ab OR ‘severe maternal morbidities’:ti,ab OR ‘severe acute maternal morbidity’:ti,ab OR ‘severe acute maternal morbidities’:ti,ab OR 'severe maternal morbidity'/exp OR 'severe acute maternal morbidity'/exp OR ‘Obstetric hemorrhage’:ti,ab OR ‘Obstetric hemorrhages’:ti,ab OR ‘Obstetric haemorrhage’:ti,ab OR ‘Obstetric haemorrhages’:ti,ab OR ‘Postpartum hemorrhage’:ti,ab OR ‘Postpartum hemorrhages’:ti,ab OR ‘Postpartum haemorrhage’:ti,ab OR ‘Postpartum haemorrhages’:ti,ab OR 'obstetric hemorrhage'/exp OR ‘peripartum hysterectomy’:ti,ab OR ‘peripartum hysterectomies’:ti,ab OR ‘peri partum hysterectomy’:ti,ab OR ‘Peri partum hysterectomies’:ti,ab OR ‘pregnancy-related hysterectomy’:ti,ab OR 'peripartum hysterectomy'/exp OR Eclampsia:ti,ab OR Eclampsias:ti,ab OR 'eclampsia'/exp OR Preeclampsia:ti,ab OR Preeclampsias:ti,ab OR ‘Pre eclampsia’:ti,ab OR ‘Pre eclampsias’:ti,ab OR ‘Pregnancy toxemia’:ti,ab OR ‘Pregnancy toxemias’:ti,ab OR ‘Toxemia of pregnancy’:ti,ab OR ‘Toxemia of pregnancies’:ti,ab OR 'preeclampsia'/exp OR ‘Maternal sepsis’:ti,ab OR 'maternal sepsis'/exp OR ‘uterine rupture’:ti,ab OR ‘uterine ruptures’:ti,ab OR 'uterine rupture in pregnancy'/exp OR ‘Maternal admission to the intensive care unit’:ti,ab OR ‘Maternal intensive care unit admission’:ti,ab OR ‘Maternal admission to an intensive care unit’:ti,ab) |
| S3 | (Maternal:ti,ab AND ‘Intensive care unit’/exp) |
| S4 | S1 AND (S2 OR S3) |
| S5 | S4 AND [1990-2023]/py |
