# Supplementary material for: Association between migration and severe maternal outcomes in high-income countries: Systematic review and meta-analysis
Source: PLoS Med. 2023 Jun 22;20(6):e1004257. doi: 10.1371/journal.pmed.1004257 (PMC10328365; doi:10.1371/journal.pmed.1004257)
Supplement: S3 Table — (DOCX) [file pmed.1004257.s003.docx]

S3 Table. Literature search algorithm on Cochrane Library

| **Set#** | **Search term** |
| --- | --- |
| S1 | (Migrant):ti,ab,kw OR (Migrants):ti,ab,kw OR (Immigrant):ti,ab,kw OR (Immigrants):ti,ab,kw OR (emigrant):ti,ab,kw OR (emigrants):ti,ab,kw OR (Foreign-born):ti,ab,kw OR (Foreign born):ti,ab,kw OR (Migration):ti,ab,kw OR (Migrations):ti,ab,kw OR (Immigration):ti,ab,kw OR (Immigrations):ti,ab,kw OR (Emigration and Immigration[mh]) OR (transients and migrants[mh]) OR (maternal country of birth):ti,ab,kw OR (maternal region of birth):ti,ab,kw OR (maternal place of birth):ti,ab,kw OR (legal status):ti,ab,kw OR (administrative status):ti,ab,kw OR (asylum seeker):ti,ab,kw OR (asylum seekers):ti,ab,kw OR (refugee):ti,ab,kw OR (refugees):ti,ab,kw OR (refugees[mh]) OR (expatriate):ti,ab,kw OR (expatriates):ti,ab,kw OR (exile):ti,ab,kw OR (exiles):ti,ab,kw |
| S2 | (maternal mortality):ti,ab,kw OR ("maternal mortalities"):ti,ab,kw OR (maternal mortality[mh]) OR ("maternal death"):ti,ab,kw OR ("maternal deaths"):ti,ab,kw OR (maternal death[mh]) OR ("Pregnancy-related mortality"):ti,ab,kw OR ("Pregnancy-related mortalities"):ti,ab,kw OR ("Pregnancy-associated mortality"):ti,ab,kw OR ("Pregnancy-associated mortalities"):ti,ab,kw OR ("Maternal near-miss"):ti,ab,kw OR ("severe maternal morbidity"):ti,ab,kw OR ("severe maternal morbidities"):ti,ab,kw OR ("severe acute maternal morbidity"):ti,ab,kw OR ("severe acute maternal morbidities"):ti,ab,kw OR ("Obstetric hemorrhage"):ti,ab,kw OR ("Obstetric hemorrhages"):ti,ab,kw OR ("Obstetric haemorrhage"):ti,ab,kw OR ("Obstetric haemorrhages"):ti,ab,kw OR ("Postpartum hemorrhage"):ti,ab,kw OR ("Postpartum hemorrhages"):ti,ab,kw OR ("Postpartum haemorrhage"):ti,ab,kw OR ("Postpartum haemorrhages"):ti,ab,kw OR ("Postpartum hemorrhage"[mh]) OR (“peripartum hysterectomy”):ti,ab,kw OR (“peripartum hysterectomies”):ti,ab,kw OR (“peri partum hysterectomy”):ti,ab,kw OR (“peri partum hysterectomies”):ti,ab,kw OR (“pregnancy-related hysterectomy”):ti,ab,kw OR (Eclampsia):ti,ab,kw OR (Eclampsias):ti,ab,kw OR (Eclampsia[mh]) OR (Preeclampsia):ti,ab,kw OR (Preeclampsias):ti,ab,kw OR (“Pre eclampsia”):ti,ab,kw OR (“Pre eclampsias”):ti,ab,kw OR (“Pregnancy toxemia”):ti,ab,kw OR (“Pregnancy toxemias”):ti,ab,kw OR (“Toxemia of pregnancy”):ti,ab,kw OR (“Toxemia of pregnancies”):ti,ab,kw OR (Preeclampsia[mh]) OR ("Maternal sepsis"):ti,ab,kw OR (“uterine rupture”):ti,ab,kw OR (“uterine ruptures”):ti,ab,kw OR (uterine rupture[mh]) OR (“Maternal admission to the intensive care unit”):ti,ab,kw OR (“Maternal intensive care unit admission”):ti,ab,kw OR (“Maternal admission to an intensive care unit”):ti,ab,kw |
| S3 | ((maternal):ti,ab,kw AND (intensive care units[mh])) |
| S4 | S1 AND (S2 OR S3) |
| S5 | S4 AND (Observational study):ti,ab,kw |
