# Supplementary material for: Association between migration and severe maternal outcomes in high-income countries: Systematic review and meta-analysis
Source: PLoS Med. 2023 Jun 22;20(6):e1004257. doi: 10.1371/journal.pmed.1004257 (PMC10328365; doi:10.1371/journal.pmed.1004257)
Supplement: S4 Table — (DOCX) [file pmed.1004257.s004.docx]

S4 Table. List of the excluded studies and reasons for their exclusion

| **Studies** | **Exclusion Reason** |
| --- | --- |
| Acunzo M., Autuori M.C., Cortinovis I., Marconi A.M. Pregnancy and birth: The role of migration. Reproductive Sciences 2018 25:1 (306A-307A) | Did not measure outcomes of interest |
| Acunzo M., Autuori M.C., Cortinovis I., Marconi A.M. Pregnancy and birth: The role of access to care. Reproductive Sciences 2018 25:1 (165A-) | Did not measure outcomes of interest |
| Adegoke T., Pinder L., Ndiwane N., Vragovic O. Adverse maternal and perinatal outcomes: A comparison of United States- and foreign-born women. Obst Gynecol 2017 129 Supplement 1 (39S-) | Did not measure outcomes of interest |
| Bains S, Mæland KS, Vik ES. Prenatal health of immigrant women in Norway - an exploratory literature review. Tidsskr Den Nor Laegeforening Tidsskr Prakt Med Ny Raekke. 2021;141. | Did not measure outcomes of interest |
| Bakken KS, Skjeldal OH, Stray-Pedersen B. Immigrants from conflict-zone countries: an observational comparison study of obstetric outcomes in a low-risk maternity ward in Norway. BMC Pregnancy Childbirth. 2015;15:163. | Did not measure outcomes of interest |
| Bakken KS, Skjeldal OH, Stray-Pedersen B. Obstetric Outcomes of First- and Second-Generation Pakistani Immigrants: A Comparison Study at a Low-Risk Maternity Ward in Norway. J Immigr Minor Health. 2017;19:33‑40. | Did not measure outcomes of interest |
| Bastola K, Koponen P, Skogberg N, Gissler M, Kinnunen TI. Hypertensive disorders of pregnancy among women of migrant origin in Finland: A population-based study. Acta Obstet Gynecol Scand. 2021. | Did not measure outcomes of interest |
| Boakye E, Sharma G, Ogunwole SM, Zakaria S, Vaught AJ, Kwapong YA, et al. Relationship of Preeclampsia With Maternal Place of Birth and Duration of Residence Among Non-Hispanic Black Women in the United States. Circ Cardiovasc Qual Outcomes. 2021;14. | Did not measure outcomes of interest |
| Borovich A., Chen R., Orbach-Zinger S., Nassie D.I., Shmueli A., Hadar E., Wiznitzer A., Krispin E. Obstetrical and perinatal outcomes among asylum seekers & work immigrants. Am J Obstet Gynecol 2018 218:1 Supplement 1 (S566-S567) | Did not measure outcomes of interest |
| Davies-Tuck ML, Davey M-A, Wallace EM. Maternal region of birth and stillbirth in Victoria, Australia 2000-2011: A retrospective cohort study of Victorian perinatal data. PLOS One. 2017;12:e0178727. | Did not measure outcomes of interest |
| Essén B, Hanson BS, Östergren P-O, Lindquist PG, Gudmundsson S. Increased perinatal mortality among sub-Saharan immigrants in a city-population in Sweden. Acta Obstet Gynecol Scand. 2000;79:737‑43. | Did not measure outcomes of interest |
| Flood M., Pollock W., McDonald S., Cullinane F., Davey M.-A. Maternal country of birth and blood transfusion for 370,603 confinements in Victoria. Women and Birth 2019 32 Supplement 1 (S14-) | Did not measure outcomes of interest |
| Forna F, Jamieson DJ, Sanders D, Lindsay MK. Pregnancy outcomes in foreign-born and US-born women. Int J Gynaecol Obstet Off Organ Int Fed Gynaecol Obstet. 2003;83:257‑65. | Did not measure outcomes of interest |
| Funai EF, Paltiel OB, Malaspina D, Friedlander Y, Deutsch L, Harlap S. Risk factors for pre-eclampsia in nulliparous and parous women: the Jerusalem perinatal study. Paediatr Perinat Epidemiol. 2005;19:59‑68. | Did not measure outcomes of interest |
| Gong J, Savitz DA, Stein CR, Engel SM. Maternal ethnicity and pre-eclampsia in New York City, 1995-2003: Pre-eclampsia and maternal ethnicity. Paediatr Perinat Epidemiol. 2012;26:45‑52. | Did not measure outcomes of interest |
| Guðmundsdóttir EÝ, Gottfreðsdóttir H, Hálfdánsdóttir B, Nieuwenhuijze M, Gissler M, Einarsdóttir K. Challenges in migrant women’s maternity care in a high‐income country: A population‐based cohort study of maternal and perinatal outcomes. Acta Obstet Gynecol Scand. 2021;aogs.14186. | Did not measure outcomes of interest |
| Harakow H-I, Hvidman L, Wejse C, Eiset AH. Pregnancy complications among refugee women: A systematic review. Acta Obstet Gynecol Scand. 2021;100:649‑57. | Did not measure outcomes of interest |
| Henry OA, Guaran RL, Petterson CD, Walstab JE. Obstetric and birthweight differences between Vietnam-born and Australian-born women. Med J Aust. 1992;156:321‑4. | Did not measure outcomes of interest |
| Kanthasamy M, Bognanno A, Subramanian V, Macneilly L, Miguel L, Dong S, et al. Obstetric outcome of ethnic Turkish women in London: a retrospective case-control study. J Obstet Gynaecol J Inst Obstet Gynaecol. 2013;33:367‑9. | Did not measure outcomes of interest |
| Khan S, Yao Z, Shah BR. Gestational diabetes care and outcomes for refugee women: a population-based cohort study. Diabet Med J Br Diabet Assoc. 2017;34:1608‑14. | Did not measure outcomes of interest |
| Kragelund Nielsen K; Andersen GS; Damm P; Andersen AN. Migration, gestational diabetes and adverse pregnancy outcomes: a nationwide study of singleton deliveries in Denmark. J Clin Endocrinol Metab 2021 | Did not measure outcomes of interest |
| Lolos M., Koutroumanis P., Daskalakis G., Loutradis D. Clinical characteristics, antenatal care and pregnancy outcomes among pregnant refugees delivering in a tertiary university hospital in Greece. Eur J Obstet Gynecol Reprod Biol 2019 234 (e141-) | Did not measure outcomes of interest |
| Ma J, Bauman A. Obstetric profiles and pregnancy outcomes of immigrant women in New South Wales, 1990-1992. Aust N Z J Obstet Gynaecol. 1996;36:119‑25. | Did not measure outcomes of interest |
| Macfarlane A., Datta-Nemdharry P., Dattani N. Reproductive and perinatal health among migrants to England and Wales. Eur J Epidemiol 2012 27:1 SUPPL. 1 (S54-) | Did not measure outcomes of interest |
| Mæland KS, Sande RK, Bing-Jonsson PC. Risk for delivery complications in Robson Group 1 for non-Western women in Norway compared with ethnic Norwegian women - A population-based observational cohort study. Sex Reprod Healthc Off J Swed Assoc Midwives. 2019;20:42‑5. | Did not measure outcomes of interest |
| Mæland KS, Morken NH, Schytt E, Aasheim V, Nilsen RM. Risk of Subsequent Preeclampsia by Maternal Country of Birth: A Norwegian Population-Based Study. Int J Environ Res Public Health. 2023;20:4109. | Did not measure outcomes of interest |
| Margioula-Siarkou C, Petousis S, Kalogiannidis I, Dagklis T, Traianos V, Goutzioulis M, et al. Immigrants present improved obstetric and neonatal outcomes compared to native women. A northern Greek population analysis. J Immigr Minor Health. 2013;15:249‑54. | Did not measure outcomes of interest |
| Mattsson K, Juárez S, Malmqvist E. Influence of Socio-Economic Factors and Region of Birth on the Risk of Preeclampsia in Sweden. Int J Environ Res Public Health. 2022;19:4080. | Did not measure outcomes of interest |
| Mumtaz Z, O’Brien B, Higginbottom G. Navigating maternity health care: a survey of the Canadian prairie newcomer experience. BMC Pregnancy Childbirth. 2014;14:4. | Did not measure outcomes of interest |
| Naimy Z, Grytten J, Monkerud L, Eskild A. The prevalence of pre-eclampsia in migrant relative to native Norwegian women: a population-based study. BJOG Int J Obstet Gynaecol. 2015;122:859‑65. | Did not measure outcomes of interest |
| Nilsen RM, Vik ES, Rasmussen SA, Small R, Moster D, Schytt E, et al. Preeclampsia by maternal reasons for immigration: a population-based study. BMC Pregnancy Childbirth. 2018;18:423. | Did not measure outcomes of interest |
| Ortiz J., Díaz M., Quiroz J., Pavez J., Carroza B., Binfa L., Araya B.M. Comparison of nutritional status Y perinatal outcomes between immigrant and chilean pregnant women who attended their delivery at San Borja Arriaran hospital in the year 2015. Annals of Nutrition and Metabolism 2017 71 Supplement 2 (1151-) | Did not measure outcomes of interest |
| Ortiz J, Diaz M, Araya B M, Quiroz J, Carroza B, Pavez J, et al. Comparison of bio-sociodemographic, obstetric and perinatal characteristics among immigrant and native women in the Metropolitan Region in Chile. Midwifery. 2019;75:72‑9. | Did not measure outcomes of interest |
| Panagopoulos P, Tsoukalos G, Economou A, Zikopoulos M, Koutras I, Petrakos G, et al. Delivery and immigration: the experience of a Greek Hospital. Clin Exp Obstet Gynecol. 2005;32:55‑7. | Did not measure outcomes of interest |
| Råssjö EB, Byrskog U, Samir R, Klingberg-Allvin M. Somali women’s use of maternity health services and the outcome of their pregnancies: a descriptive study comparing Somali immigrants with native-born Swedish women. Sex Reprod Health Off J Swed Assoc Midwives. 2013;4:99‑106. | Did not measure outcomes of interest |
| Ray JG, Wanigaratne S, Park AL, Bartsch E, Dzakpasu S, Urquia ML. Preterm preeclampsia in relation to country of birth. J Perinatol Off J Calif Perinat Assoc. 2016;36:718‑22. | Did not measure outcomes of interest |
| Shah D, Tay A, Desai A, Parikh M, Nauta M, Yoong W. The obstetric performance of Chinese immigrants residing in the UK. J Obstet Gynaecol J Inst Obstet Gynaecol. 2011;31:480‑2. | Did not measure outcomes of interest |
| Sharma G., Boakye E., Dzaye O., Kwapong Y.A., Zakaria S., Vaught A., Creanga A., Cainzos-Achirica M., Mehta L., Nasir K., Blaha M., Blumenthal R., Wang X. THE ASSOCIATION OF PREECLAMPSIA AND CARDIOVASCULAR RISK FACTORS WITH MATERNAL NATIVITY AND DURATION OF US RESIDENCE AMONG TWO DIVERSE RACIAL GROUPS. J Am Coll Cardiol 2021 77:18 Supplement 1 (3394-) | Did not measure outcomes of interest |
| Sole KB, Staff AC, Laine K. The association of maternal country of birth and education with hypertensive disorders of pregnancy: A population-based study of 960 516 deliveries in Norway. Acta Obstet Gynecol Scand. 2018;97:1237‑47. | Did not measure outcomes of interest |
| Sullivan JR, Shepherd SJ. Obstetric outcomes and infant birthweights for Vietnamese-born and Australian-born women in southwestern Sydney. Aust N Z J Public Health. 1997;21:159‑62. | Did not measure outcomes of interest |
| Tay A., Desai A., Parikh M., Melendez J., Nauta M., Fakokunde A., Yoong W. The obstetric outcomes of Chinese immigrants residing in the United Kingdom: A case control study. International Journal of Gynecology and Obstetrics 2009 107 SUPPL. 2 (S264-) | Did not measure outcomes of interest |
| Theodora M., Antsaklis P., Michala L., Lolos M., Kalambalikis A., Koutroumanis P., Loutradis D., Daskalakis G. Perinatal outcomes among immigrants and refugees in comparison with Greek population in a Tertiary University Hospital in Greece. Journal of Perinatal Medicine 2019 47 Supplement 1 (eA146-) | Did not measure outcomes of interest |
| Thomas J.W., Judkins A. Increased rate of chorioamnionitis among pregnant immigrant women living in utah. Journal of Investigative Medicine 2019 67:1 (118-) | Did not measure outcomes of interest |
| Uppal P, Holland AJA, Bajuk B, Abdel-Latif M, Jaffe A, Hilder L, et al. The association between maternal country of birth and neonatal intensive care unit outcomes. Early Hum Dev. 2013;89:607‑14. | Did not measure outcomes of interest |
| Van Oostrum IEA, Goosen S, Uitenbroek DG, Koppenaal H, Stronks K. Mortality and causes of death among asylum seekers in the Netherlands, 2002-2005. J Epidemiol Community Health. 2011;65:376‑83. | Did not measure outcomes of interest |
| Vangen S, Stoltenberg C, Holan S, Moe N, Magnus P, Harris JR, et al. Outcome of pregnancy among immigrant women with diabetes. Diabetes Care. 2003;26:327‑32. | Did not measure outcomes of interest |
| von Katterfeld B, Li J, McNamara B, Langridge AT. Obstetric profiles of foreign-born women in Western Australia using data linkage, 1998-2006. Aust N Z J Obstet Gynaecol. 2011;51:225‑32. | Did not measure outcomes of interest |
| von Katterfeld B, Li J, McNamara B, Langridge AT. Maternal and neonatal outcomes associated with gestational diabetes in women from culturally and linguistically diverse backgrounds in Western Australia. Diabet Med J Br Diabet Assoc. 2012;29:372‑7. | Did not measure outcomes of interest |
| Wasse H, Holt VL, Daling JR. Pregnancy risk factors and birth outcomes in Washington State: a comparison of Ethiopian-born and US-born women. Am J Public Health. 1994;84:1505‑7. | Did not measure outcomes of interest |
| Westerway SC, Keogh J, Heard R, Morris J. Incidence of fetal macrosomia and birth complications in Chinese immigrant women. Aust N Z J Obstet Gynaecol. 2003;43:46‑9. | Did not measure outcomes of interest |
| Wildsmith EM. Testing the weathering hypothesis among Mexican-origin women. Ethn Dis. 2002;12:470‑9. | Did not measure outcomes of interest |
| Yim C, Wong L, Cabalag C, Wallace EM, Davies-Tuck M. Post-term surveillance and birth outcomes in South Asian-born compared with Australian-born women. J Perinatol Off J Calif Perinat Assoc. 2017;37:139‑43. | Did not measure outcomes of interest |
| Yoong W, Wagley A, Fong C, Chukwuma C, Nauta M. Obstetric performance of ethnic Kosovo Albanian asylum seekers in London: a case – control study. J Obstet Gynaecol. 2004;24:510‑2. | Did not measure outcomes of interest |
| Vangen S, Stoltenberg C, Johansen REB, Sundby J, Stray-Pedersen B. Perinatal complications among ethnic Somalis in Norway. Acta Obstet Gynecol Scand. 2002;81:317‑22. | Did not measure outcomes of interest |
| Kuvacic I, Skrablin S, Hodzic D, Milkovic G. Possible influence of expatriation on perinatal outcome. Acta Obstet Gynecol Scand. 1996;75:367‑71. | Did not measure outcomes of interest |
| Small R, Gagnon A, Gissler M, Zeitlin J, Bennis M, Glazier R, et al. Somali women and their pregnancy outcomes postmigration: data from six receiving countries. BJOG Int J Obstet Gynaecol. 2008;115:1630‑40. | Did not measure outcomes of interest |
| Bray JK, Gorman DR, Dundas K, Sim J. Obstetric care of new European migrants in Scotland: an audit of antenatal care, obstetric outcomes and communication. Scott Med J. 2010;55:26‑31. | Did not measure outcomes of interest |
| van Roosmalen J, Schuitemaker NWE, Brand R, van Dongen PWJ, Bennebroek Gravenhorst J. Substandard care in immigrant versus indigenous maternal deaths in The Netherlands. BJOG Int J Obstet Gynaecol. 2002;109:212‑3. | Did not measure outcomes of interest |
| Urquia ML, O’Campo PJ, Heaman MI. Revisiting the immigrant paradox in reproductive health: the roles of duration of residence and ethnicity. Soc Sci Med 1982. 2012;74:1610‑21. | Did not measure outcomes of interest |
| Verschuuren AEH, Postma IR, Riksen ZM, Nott RL, Feijen-de Jong EI, Stekelenburg J. Pregnancy outcomes in asylum seekers in the North of the Netherlands: a retrospective documentary analysis. BMC Pregnancy Childbirth. 2020;20:320. | Did not measure outcomes of interest |
| Abouzahr C. Maternal mortality -- how big a problem do I have and how can I measure it? Safe Mother. 1997;(23):12‑3. | Definition of migrant women not based on region of birth |
| Al-Rubaie ZTA, Malcolm Hudson H, Jenkins G, Mahmoud I, Ray JG, Askie LM, et al. The association between ethnicity and pre-eclampsia in Australia: A multicentre retrospective cohort study. Aust N Z J Obstet Gynaecol. 2020;60:396‑404. | Definition of migrant women not based on region of birth |
| Arkansas Maternal Mortality Review Committee Members. Arkansas maternal mortlaity review. 2020 | Definition of migrant women not based on region of birth |
| Armbrust R, von Rennenberg R, David M. A Retrospective Perinatal Data Analysis of Immigrant and German Women from Representative Birth Cohorts at the Virchow Hospital, Berlin. Geburtshilfe Frauenheilkd. 2016;76:1157‑62. | Definition of migrant women not based on region of birth |
| Bardin L, Schiffmacher A, Ricketts S, et al. Understanding maternal deaths in Colorado : an analysis of mortality from 2008-2013. 2017 | Definition of migrant women not based on region of birth |
| Bollini P, Wanner P, Pampallona S. Trends in maternal mortality in Switzerland among Swiss and foreign nationals, 1969-2006. Int J Public Health. 2011;56:515‑21. | Definition of migrant women not based on region of birth |
| Brehm Christensen M, Fredsted Villadsen S, Weber T, Wilken-Jensen C, Nybo Andersen A-M. Higher rate of serious perinatal events in non-Western women in Denmark. Dan Med J. 2016;63:A5197. | Definition of migrant women not based on region of birth |
| Building U.S. Capacity to Review and Prevent Maternal Deaths. (2017). Report from maternal mortality review committees : a view into their critical role. | Definition of migrant women not based on region of birth |
| Building U.S. Capacity to Review and Prevent Maternal Deaths. (2018). Report from nine maternal mortality review committees. Retrieved from http://reviewtoaction.org/Report_from_Nine_MMRCs | Definition of migrant women not based on region of birth |
| Cabasag C, Rubio V, Williams D et al. Arizona Maternal Mortality Review Program 2012-2015. 2019 | Definition of migrant women not based on region of birth |
| California Pregnancy Mortality Surveillance System. California Pregnancy-Related Deaths, 2008-2016. 2021 | Definition of migrant women not based on region of birth |
| Cho GJ, Kim HY, Ko HS, Cho HJ, Hong SY, Noh E, et al. Pregnancy outcomes of immigrant women living in Korea: A population-based study. PLOS One. 2022;17:e0278193 | Definition of migrant women not based on region of birth |
| Connecticut Maternal Mortality review commitee. Maternal Mortality in Connecticut, 2015-2017. 2020 | Definition of migrant women not based on region of birth |
| David M, Pachaly J, Vetter K. Perinatal outcome in Berlin (Germany) among immigrants from Turkey. Arch Gynecol Obstet. 2006;274:271‑8. | Definition of migrant women not based on region of birth |
| Davis G, Jellins J. Female genital mutilation: Obstetric outcomes in metropolitan Sydney. Aust N Z J Obstet Gynaecol. 2019;59:312‑6. | Definition of migrant women not based on region of birth |
| Donati S, Senatore S, Ronconi A, Regional Maternal Mortality Working Group. Obstetric near-miss cases among women admitted to intensive care units in Italy. Acta Obstet Gynecol Scand. 2012;91:452‑7. | Definition of migrant women not based on region of birth |
| Donati S, Maraschini A, Lega I, D’Aloja P, Buoncristiano M, Manno V, et al. Maternal mortality in Italy: Results and perspectives of record-linkage analysis. Acta Obstet Gynecol Scand. 2018;97:1317‑24. | Definition of migrant women not based on region of birth |
| Esscher A, Binder-Finnema P, Bodker B, Hogberg U, MulicLutvica A, Essen B. Suboptimal care and maternal mortality among foreign-born women in Sweden: maternal death audit with application of the ‘migration three delays’ model. BMC Pregnancy Childbirth. 2014;14:141. | Definition of migrant women not based on region of birth |
| Fasanya HO, Hsiao CJ, Armstrong-Sylvester KR, Beal SG. A Critical Review on the Use of Race in Understanding Racial Disparities in Preeclampsia. J Appl Lab Med. 2021;6:247‑56. | Definition of migrant women not based on region of birth |
| Gudmundsdottir EY, Vigfusdottir L, Gottfredsdottir H. [Preterm birth among Icelandic and migrant women in Iceland during 1997-2018 and main contributing factors]. Laeknabladid. 2023;109:75‑81. | Definition of migrant women not based on region of birth |
| Hanprasertpong T, Hanprasertpong J. Pregnancy outcomes in Southeast Asian migrant workers at Southern Thailand. J Obstet Gynaecol J Inst Obstet Gynaecol. 2015;35:565‑9. | Definition of migrant women not based on region of birth |
| Hernandez L, Thompson A, et al. Florida’s Maternal Mortality Review Committee 2019. 2021 | Definition of migrant women not based on region of birth |
| Howell EA, Egorova NN, Janevic T, Balbierz A, Zeitlin J, Hebert PL. Severe Maternal Morbidity Among Hispanic Women in New York City: Investigation of Health Disparities. Obstet Gynecol. 2017;129:285‑94. | Definition of migrant women not based on region of birth |
| Indiana maternal mortality review committee : 2020 annual report. 2020 | Definition of migrant women not based on region of birth |
| Kieltyka L, Mehta P, Schoellmann K, Lake C, Zapata A, Herwege J et al. Louisiana maternal mortality review report 2011-2016. 2018 | Definition of migrant women not based on region of birth |
| Knight M, Kurinczuk JJ, Spark P, Brocklehurst P, UKOSS. Inequalities in maternal health: national cohort study of ethnic variation in severe maternal morbidities. BMJ. 2009;338:b542. | Definition of migrant women not based on region of birth |
| Knupp R.J., Yuanfan Y., Szychowski J.M., Battarbee A.N., Subramaniam A. 234 Adverse maternal and neonatal outcomes in latinas compared to Non-Latinx black and Non-Latinx white women. Am J Obstet Gynecol 2021 224:2 Supplement (S154-S155) | Definition of migrant women not based on region of birth |
| Lewis G.K., Dawkins J.C., Boddu R., Cabral P.A. Obstetric Outcomes in Nepali Immigrant Patients: A Retrospective Study Within the Rochester Regional Health System. Obstet and Gynecol 2019 133:SUPPL 1 | Definition of migrant women not based on region of birth |
| Maternal Mortality and Morbidity Review Committee. Texas Maternal Mortality and Morbidity Review Committee and Department of State Health Services Joint Biennial Report. 2020 | Definition of migrant women not based on region of birth |
| Maternal Mortality Review Committee. Illinois Maternal Morbidity and Mortality Report 2016-2017. 2021 | Definition of migrant women not based on region of birth |
| McDonald JA, Rishel K, Escobedo MA, Arellano DE, Cunningham TJ. Obstetric emergencies at the United States-Mexico border crossings in El Paso, Texas. Rev Panam Salud Publica Pan Am J Public Health. 2015;37:76‑82. | Definition of migrant women not based on region of birth |
| Mckitt T and the Alabama Maternal Mortality Review Committee. Review of 2016 maternal mortality. 2016 | Definition of migrant women not based on region of birth |
| Salim R, Mfra A, Garmi G, Shalev E. Comparison of intrapartum outcome among immigrant women from Ethiopia and the general obstetric population in Israel. Int J Gynaecol Obstet Off Organ Int Fed Gynaecol Obstet. 2012;118:161‑5. | Definition of migrant women not based on region of birth |
| The Infant and Maternal Mortality review panel. West Virginia infant and maternal mortality review panel : annual report 2011. 2013 | Definition of migrant women not based on region of birth |
| The Kansas Maternal Mortality Review Committee. Kansas Maternal mortality report 2016-2018. 2020 | Definition of migrant women not based on region of birth |
| The Latin America and Caribbean Task Force for Maternal Mortality Reduction. Overview of the Situation of Maternal Morbidity and Mortality: Latin America and the Caribbean. 2017 | Definition of migrant women not based on region of birth |
| The Mississippi maternal mortality review committee. Mississippi maternal mortality report 2013-2016. 2019 | Definition of migrant women not based on region of birth |
| The Oregon maternal mortality and morbidity review committee. Oregon maternal mortality and morbidity review committee biennial report. 2021 | Definition of migrant women not based on region of birth |
| The Perinatal and Maternal Mortality Review Committee. Fourteenth Annual Report of the Perinatal and Maternal Mortality Review Committee. 2021 | Definition of migrant women not based on region of birth |
| The South Dakota Advisory Committee. Maternal Mortality and Health disparities of American Indian Women in South Dakota. 2021 | Definition of migrant women not based on region of birth |
| Almeida LM, Caldas J, Ayres-de-Campos D, Salcedo-Barrientos D, Dias S. Maternal healthcare in migrants: a systematic review. Matern Child Health J. 2013;17:1346‑54. | Non-original data |
| Ayuso Herrera E, Alonso Mayo C, Garcia-Tizon Larroca S. Maternal Mortality Among Immigrant Women in Europe and the USA: a Systematic Review. SN Compr Clin Med. 2020;2:16‑24. | Non-original data |
| Bloch JR. Postpartum health, service needs, and access to care experiences of immigrant and Canadian-born women. J Obstet Gynecol Neonatal Nurs JOGNN. 2007;36:172‑3; author reply 173. | Non-original data |
| Bragg R. Maternal deaths and vulnerable migrants. Lancet Lond Engl. 2008;371:879‑81. | Non-original data |
| García-Tizón Larroca S, Amor Valera F, Ayuso Herrera E, Cueto Hernandez I, Cuñarro Lopez Y, De Leon-Luis J. Human Development Index of the maternal country of origin and its relationship with maternal near miss: A systematic review of the literature. BMC Pregnancy Childbirth. 2020;20:224. | Non-original data |
| Gieles NC, Tankink JB, van Midde M, Düker J, van der Lans P, Wessels CM, et al. Maternal and perinatal outcomes of asylum seekers and undocumented migrants in Europe: a systematic review. Eur J Public Health. 2019;29:714‑23. | Non-original data |
| Hamlyn J, Duhig M, McGrath J, Scott J. Modifiable risk factors for schizophrenia and autism--shared risk factors impacting on brain development. Neurobiol Dis. mai 2013;53:3‑9. | Non-original data |
| Heslehurst N, Brown H, Pemu A, Coleman H, Rankin J. Perinatal health outcomes and care among asylum seekers and refugees: a systematic review of systematic reviews. BMC Med. 2018;16:89. | Non-original data |
| López-Quesada E., Prada E. Immigration obstetrics in a regional hospital. Gestational morbidity: Pathology of immigration-associated pregnancy. Ginecologia y Obstetricia Clinica 2005 6:1 (8-10) | Non-original data |
| Mogos MF, Salinas-Miranda AA, Salemi JL, Medina IM, Salihu HM. Pregnancy-Related Hypertensive Disorders and Immigrant Status: A Systematic Review and Meta-analysis of Epidemiological Studies. J Immigr Minor Health. 2017;19:1488‑97. | Non-original data |
| Mogos M.F., Salinas-Miranda A.A., Salihu H.M., Medina I.M., Salemi J.L. Pre-eclampsia and immigrant status: A systematic review and meta-analysis of epidemiological studies. Reproductive Sciences 2016 23:1 SUPPL. 1 (301A-) | Non-original data |
| Monga M. Obstetric Outcome: The Immigrant Effect? Crit Care Med. 2016;44:1430‑1. | Non-original data |
| Morgan SA, Ali MM. A review of methodology and tools for measuring maternal mortality in humanitarian settings. Health Policy Plan. 2018;33:1107‑17. | Non-original data |
| Ogah OS, Rayner BL. Recent advances in hypertension in sub-Saharan Africa. Heart Br Card Soc. 2013;99:1390‑7. | Non-original data |
| Pedersen GS, Grøntved A, Mortensen LH, Andersen A-MN, Rich-Edwards J. Maternal mortality among migrants in Western Europe: a meta-analysis. Matern Child Health J. 2014;18:1628‑38. | Non-original data |
| Pedersen G.S., Grøntved A., Mortensen L.H., Andersen A.-M.N., Rich-Edwards J.W. Immigrant women and maternal mortality in Western Europe: A meta-analysis of observational studies. Eur J Epidemiol 2013 28:1 SUPPL. 1 (S213-) | Non-original data |
| Pedersen G.S., Grontved A., Mortensen L.H., Andersen A.-M.N., Rich-Edwards J.W. Immigrant women and maternal mortality in Western Europe: A meta-analysis of observational studies. Am J Epidemiol 2012 175 SUPPL. 11 (S134-) | Non-original data |
| Schuitemaker NW. Maternal mortality in Europe; present and future. Eur J Obstet Gynecol Reprod Biol. 1999;86:129‑30. | Non-original data |
| Blagoeva Atanasova V, Arevalo-Serrano J, Antolin Alvarado E, García-Tizón Larroca S. Maternal mortality in Spain and its association with country of origin: cross-sectional study during the period 1999-2015. BMC Public Health. 2018;18:1171. | Duplicate studies |
| Deneux-tharaux C., Saucedo M. Epidemiology of maternal mortality in France, 2010–2012. Anesthesie et Reanimation 2018 4:1 (16-35) | Duplicate studies |
| Eslier M, Sauvegrain P, Estellat C, Schmitz T, Luton D, Mandelbrot L, et al. 863: Association between undocumented immigrant status and prenatal care utilization, severe perinatal and maternal morbidity. Am J Obstet Gynecol. 2020;222:S539. | Duplicate studies |
| Luque Fernández MÁ, Cavanillas AB, de Mateo S. Excess of maternal mortality in foreign nationalities in Spain, 1999–2006. Eur J Obstet Gynecol Reprod Biol. mars 2010;149:52‑6. | Duplicate studies |
| Luque Fernández MÁ, Gutiérrez Garitano I, Cavanillas AB. Increased risk of maternal deaths associated with foreign origin in Spain: a population based case-control study. Eur J Public Health. 2011;21:292‑4. | Duplicate studies |
| Mujahid MS, Kan P, Leonard SA, Hailu EM, Wall-Wieler E, Abrams B, et al. Birth hospital and racial and ethnic differences in severe maternal morbidity in the state of California. Am J Obstet Gynecol. 2021;224:219.e1-219.e15. | Duplicate studies |
| Nilsen R.M., Vik E.S., Rasmussen S.A., Small R., Moster D., Schytt E., Aasheim V. Preeclampsia by maternal reasons for immigration: A population-based study 11 Medical and Health Sciences 1114 Paediatrics and Reproductive Medicine. BMC Pregnancy and Childbirth 2018 18:1 Article Number 423 | Duplicate studies |
| Reime B., Janssen P.A., Farris L., Hellmers C., Myezwa H., Borde T., Wenzlaff P. Severe acute maternal morbidity (SAMM) among migrant women in Germany. Tropical Medicine and International Health 2011 16 SUPPL. 1 (95-) | Duplicate studies |
| Schutte J.M., Steegers E.A.P., Schuitemaker N.W.E., Santema J.G., De Boer K., Pel M., Vermeulen G., Visser W., Van Roosmalen J. Rise in maternal mortality in the Netherlands. Obstetrical and Gynecological Survey 2010 65:6 (364-365) | Duplicate studies |
| Schutte J., Steegers E., Schuitemaker N., Santema J., De Boer K., Pel M., Vermeulen G., Roosmalen J.V. Rise in maternal mortality in The Netherlands. International Journal of Gynecology and Obstetrics 2009 107 SUPPL. 2 (S331-) | Duplicate studies |
| Sharma G., Boakye E., Creanga A.A., Vaught A.J., Zakaria S., Mehta L.S., Blumenthal R.S., Hong X., Ogunwole S.M., Ji Y., Wang X. The Relationship of Preeclampsia and Maternal Region of Birth and Duration of Residence in Non-US Born Black Women. Circulation 2020 142:SUPPL 3 | Duplicate studies |
| Singh GK, Lee H. Trends and Racial/Ethnic, Socioeconomic, and Geographic Disparities in Maternal Mortality from Indirect Obstetric Causes in the United States, 1999-2017. Int J MCH AIDS. 2021;10:43‑54. | Duplicate studies |
| Zwart J.J., Van Roosmalen J.J.M. Severe acute maternal morbidity in the netherlands: The lemmon study. Journal of Maternal-Fetal and Neonatal Medicine 2012 25 SUPPL. 2 (20-) | Duplicate studies |
| Zwart J., Jonkers M., Richters A., Ory F., Bloemenkamp K., Duvekot H., Van Roosmalen J. Ethnic differences in pregnancy-related severe maternal morbidity: A nationwide cohort study in the Netherlands. International Journal of Gynecology and Obstetrics 2009 107 SUPPL. 2 (S396-) | Duplicate studies |
| Garcia-Tizon Larroca S, Arevalo-Serrano J, Duran Vila A, Pintado Recarte MP, Cueto Hernandez I, Solis Pierna A, et al. Human Development Index (HDI) of the maternal country of origin as a predictor of perinatal outcomes - a longitudinal study conducted in Spain. BMC Pregnancy Childbirth. 2017;17:314. | Host country women were not the comparison group |
| Haelterman E, Qvist R, Barlow P, Alexander S. Social deprivation and poor access to care as risk factors for severe pre-eclampsia. Eur J Obstet Gynecol Reprod Biol. 2003;111:25‑32. | Host country women were not the comparison group |
| Mitsiakos G., Gialamprinou D., Chatzigrigoriou F., Karagkiozi A., Chatziioannidis I., Papacharalampous E., Karagianni P., Soubasi V. Epidimiological study of neonatal refugees in Greece. Journal of Perinatal Medicine 2019 47 Supplement 1 (eA283-eA284) | Host country women were not the comparison group |
| Ray JG, Vermeulen MJ, Schull MJ, Singh G, Shah R, Redelmeier DA. Results of the Recent Immigrant Pregnancy and Perinatal Long-term Evaluation Study (RIPPLES). CMAJ Can Med Assoc J J Assoc Medicale Can. 2007;176:1419‑26. | Host country women were not the comparison group |
| Segal S, Gemer O, Yaniv M. The outcome of pregnancy in an immigrant Ethiopian population in Israel. Arch Gynecol Obstet. 1996;258:43‑6. | Host country women were not the comparison group |
| Urquia ML, Ying I, Glazier RH, Berger H, De Souza LR, Ray JG. Serious preeclampsia among different immigrant groups. J Obstet Gynaecol Can JOGC J Obstet Gynecol Can JOGC. 2012;34:348‑52. | Host country women were not the comparison group |
| Wilson-Mitchell K, Rummens JA. Perinatal outcomes of uninsured immigrant, refugee and migrant mothers and newborns living in Toronto, Canada. Int J Environ Res Public Health. 2013;10:2198‑213. | Host country women were not the comparison group |
| Kandasamy T, Cherniak R, Shah R, Yudin MH, Spitzer R. Obstetric risks and outcomes of refugee women at a single centre in Toronto. J Obstet Gynaecol Can. 2014;36:296‑302. | Host country women were not the comparison group |
| Reed MM, Westfall JM, Bublitz C, Battaglia C, Fickenscher A. Birth outcomes in Colorado’s undocumented immigrant population. BMC Public Health. 2005;5:100. | Host country women were not the comparison group |
| Burnham G. Maternal deaths among Afghan refugees. Lancet Lond Engl. 2002;359:639‑40. | Did not take place in a high-income country |
| Hodorogea S, Friptu V. The Moldovan experience of maternal death reviews. BJOG Int J Obstet Gynaecol. 2014;121 Suppl 4:81‑5. | Did not take place in a high-income country |
| Hynes M, Sakani O, Spiegel P, Cornier N. A study of refugee maternal mortality in 10 countries, 2008-2010. Int Perspect Sex Reprod Health. 2012;38:205‑13. | Did not take place in a high-income country |
| Hynes M, Sheik M, Wilson HG, Spiegel P. Reproductive health indicators and outcomes among refugee and internally displaced persons in postemergency phase camps. JAMA. 2002;288:595‑603. | Did not take place in a high-income country |
| Guendelman S, Thornton D, Gould J, Hosang N. Mexican women in California: differentials in maternal morbidity between foreign and US-born populations. Paediatr Perinat Epidemiol. 2006;20:471‑81. | Did not provide sufficient information to calculate rates |
