# Supplementary material for: Association between migration and severe maternal outcomes in high-income countries: Systematic review and meta-analysis
Source: PLoS Med. 2023 Jun 22;20(6):e1004257. doi: 10.1371/journal.pmed.1004257 (PMC10328365; doi:10.1371/journal.pmed.1004257)
Supplement: S5 Table — (DOCX) [file pmed.1004257.s005.docx]

S5 Table. Characteristics of the selected papers, listed in alphabetical order of first authors

|  | **Country** | **Study population** | **Inclusion period** | **Study design** | **Definition of migrant women** | **Source**  **Exposure of interest** | **Reported outcomes** | **Definition of outcome** | **Source**  **Outcome** | **Total population** | **Number of migrant women** | **Migrant women's place of birth** |
| --- | --- | --- | --- | --- | --- | --- | --- | --- | --- | --- | --- | --- |
| Creanga et al. 2012 (1) | United States of America | National | 1993-2006 | Cross-sectional study | Place of birth and race/ethnicity | Birth and/or death certificates | Maternal mortality | Clinical | Medico-administrative data | 55 557 418 | 11 970 072 | Not specified |
| David et al. 2019 (2) | Germany | Multicentre | 2011-2012 | Cohort Study | Place of birth | Interview | Near-miss | Clinical | Medical file | 7100 | 2647 | Not specified |
| Deneux-Tharaux et al. 2017 (3) | France | National | 2010-2012 | Cross-sectional study | Place of birth | Birth and/or death certificates | Maternal mortality | Clinical | Medical file | 2 477 240 | 463 618 | Europe without France (15.0%)  North Africa (37.9%)  sub-Saharan Africa (21.6%)  Others (25.5%) |
| Diguisto et al. 2022 (4) | Denmark | National | 2013-2017 | Cross-sectional study | Place of birth | Birth and/or death certificates | Maternal mortality | Clinical | Medical file | 285 026 | 51976 | Not specified |
|  | Norway | National | 2014-2018 | Cross-sectional study | Place of birth | Birth and/or death certificates | Maternal mortality | Clinical | Medical file | 292 315 | 88557 | Not specified |
| Eslier et al. 2020 (5) | France | Single-center | 2008 & 2014 | Cohort Study | Place of birth | Birth and/or death certificates | Severe preeclampsia, Severe PPH, Uterine rupture | Clinical | Medical file | 6039 | 657 | Europe excluding France (16.3%)  North Africa (28.8%)  Sub-saharan Africa (30.7%)  Other (24.2%) |
| Eslier et al. 2022 (6)  Siddiqui et al. 2020 (7) | France | Multicenter | 2010-2012 | Cohort Study | Place of birth and legal status | Self-administered questionnaire | All-cause severe maternal morbidity, Maternal ICU admission, Severe preeclampsia, Eclampsia, Severe PPH, Uterine rupture, Severe sepsis | Clinical | Medical file | 9599 | 5076 | Europe (9.2%)  North Africa (41.8%)  Sub-Saharan Africa (30.9%)  Asia - Middle East (12.2%)  Others (5.8%) |
| Esscher et al. 2013 (8) | Sweden | National | 1988-2007 | Cross-sectional study | Place of birth | Birth and/or death certificates | Maternal mortality | Codes | Medical file | 2 020 091 | 325 052 | Not specified |
| Flood et al. 2020 (9) | Australia | Regional | 2009-2013 | Cohort Study | Place of birth | Birth and/or death certificates | Severe PPH | Clinical | Medico-administrative data | 364 706 | 119 238 | Americas (4.0%)  Middle East & North Africa (9.6%)  Northeast Asia (11.4%)  Northwestern Europe (9.5%)  Oceania and Antarctica (8.6%)  Southeast Asia (18.5%)  Southern and Central Asia (23.3%)  Southern and Eastern Europe (6.2%)  Sub-Saharan Africa (6.2%)  Not reported (2.7%) |
| García‑Tizón Larroca et al. 2022 (10) | Spain | National | 2000-2018 | Cross-sectional study | Place of birth | Birth and/or death certificates | Maternal mortality | Codes | Medico-administrative data | 8 439 324 | 1 619 405 | Europe (85.7%)  America (7.4%)  Africa (5.6%)  Asia (1.3%) |
| Gulersen et al. 2022 (11) | United States of America | National | 2016-2019 | Cohort Study | Place of birth | Birth register | Maternal ICU admission | Codes | Medico-administrative data | 5 576 656 | 2 028 260 | Not specified |
| Humphrey et al. 2015 (12) | Australia | National | 2008-2012 | Cross-sectional study | Place of birth | Birth and/or death certificates | Maternal mortality | Clinical | Medical file | 1 486 110 | 415 274 | Not specified |
| Humphrey et al. 2017 (13) | Australia | National | 2012-2014 | Cross-sectional study | Place of birth | Birth and/or death certificates | Maternal mortality | Clinical | Medical file | 920 095 | 290 322 | Not specified |
| Humphrey et al. 2020 (14) | Australia | National | 2015-2017 | Cross-sectional study | Place of birth | Birth and/or death certificates | Maternal mortality | Clinical | Medical file | 915 610 | 315 790 | Not specified |
| Jairam et al. 2023 (15) | Canada | Regional | 2002-2019 | Cohort study | Place of birth | Birth and/or death certificates | All-cause severe maternal morbidity | Codes | Medico-administrative data | 414 337 | 148085 | Caribbean (8.1%)  East Asia and Pacific (23.8%)  Latin America (6.5%)  Middle East and North Africa (9.3%)  South Asia (35.4%)  Sub-Saharan Africa (8.0%)  Western nations and Europe (8.8%) |
| Johnson et al. 2014 (16) | Australia | National | 2006-2010 | Cross-sectional study | Place of birth | Birth and/or death certificates | Maternal mortality | Clinical | Medical file | 1 448 445 | 368 947 | Not specified |
| Kallianidis et al. 2022 (17) | Netherlands | National | 2006-2018 | Cross-sectional study | Place of birth | Birth and/or death certificates | Maternal mortality | Codes | Medical file | 2 464 662 | 820068 | Western (28.2%)  Non-western (71.8%)  Surinam/Dutch Antilles (10.8%)  African (19.1%)  Turkey (9.7%)  Morocco (12.2%)  Asian (16.2%) |
| Knight et al. 2015 (18) | United Kingdom | National | 2011-2013 | Cross-sectional study | Place of birth | Birth and/or death certificates | Maternal mortality | Clinical | Medical file | 2 373 213 | 568 452 | Bangladesh (4.3%)  India (7.7%)  Pakistan (9.9%)  Democratic Republic of Congo (0.3%)  Nigeria (4.0%)  Somalia (2.8%)  Poland (11.4%) |
| Knight et al. 2017 (19) | United Kingdom | National | 2013-2015 | Cross-sectional study | Place of birth | Birth and/or death certificates | Maternal mortality | Clinical | Medical file | 2 305 920 | 583 114 | Bangladesh (4.1%)  Pakistan (9.6%)  Jamaica (1.0%)  Nigeria (3.8%)  Poland (12.7%) |
| Knight et al. 2020 (20) | United Kingdom | National | 2016-2018 | Cross-sectional study | Place of birth | Birth and/or death certificates | Maternal mortality | Clinical | Medical file | 2 235 159 | 604 363 | Bangladesh (3.7%)  China (1.7%)  India (6.8%)  Nigeria (3.2%)  Romania (6.9%) |
| Leonard et al. 2021 (21) | United States of America | Regional | 2011-2017 | Cohort Study | Place of birth | Birth register | All-cause severe maternal morbidity | Codes | Medico-administrative data | 3 308 554 | 1 233 767 | Not specified |
| Medcalf et al. 2016 (22) | Canada | Regional | 2003-2012 | Cohort Study | Place of birth | Birth and/or death certificates | Maternal ICU admission | Codes | Medico-administrative data | 825 827 | 305 547 | Europe and western nations (15.5%)  Middle East and North Africa (10.2%)  South Asia (31.4%)  East Asia (22.5%)  Latin America (7.6%)  Africa (7.2%)  Caribbean (5.6%) |
| Mujahid et al. 2020 (23)  Wall-Wieler et al. 2020 (24) | United States of America | Regional | 2007-2012  1997-2012 | Cohort Study | Place of birth | Birth and/or death certificates | All-cause severe maternal morbidity | Codes | Medico-administrative data | 3 010 525 | 847 379 | Not specified |
| Reime et al. 2012 (25) | Germany | Regional | 2001-2007 | Cohort Study | Place of birth or citizenship or country of residence | Birth register | Near-miss, Severe sepsis, Eclampsia, Severe PPH | Codes | Birth register | 456 061 | 74 531 | Not specified |
| Saucedo et al. 2021 (26) | France | National | 2013-2015 | Cross-sectional study | Place of birth | Birth and/or death certificates | Maternal mortality | Clinical | Medical file | 2 435 583 | 512 702 | Europe (17.8%)  North Africa (37.2%)  Sub-Saharan Africa (24.4%)  Others (20.6 %) |
| Schutte et al. 2009 (27) | Netherlands | National | 1996-2005 | Cross-sectional study | Place of birth | Birth and/or death certificates | Maternal mortality | Codes | Medical file | 2 269 506 | 753 174 | Western migrant women (21.9%)  Non-Western migrant women (39.1%)  Surinam/Dutch Antilles (8.6%)  Turkey (8.9%)  Morocco (9.5%)  Other migrant women (12.0%) |
| Singh et al. 2021 (28) | United States of America | National | 2013-2017 | Cross-sectional study | Place of birth | Birth and/or death certificates | Maternal mortality | Codes | Medico-administrative data | 19 704 375 | 4 443 057 | Not specified |
| Turner et al. 2020 (29) | Australia | Single-center | 2007-2018 | Cohort Study | Legal status | Birth and/or death certificates | Maternal ICU admission, Severe PPH | Codes | Medico-administrative data | 55 404 | 2750 | Not specified |
| Urquia et al. 2014 (30) | Multi-country (Australia,  Canada,  Spain,  United States,  Denmark, Sweden) | National or Regional | 1995-2010 | Cohort Study | Place of birth | Birth and/or death certificates | Eclampsia | Codes | Medico-administrative data | 9 028 802 | 3 031 399 | Eastern Europe (3.0%)  Western Europe (6.1%)  Latin America & the Caribbean (57.0%)  Middle East & North Africa & (6.8%)  Sub-Saharan Africa (3.2%)  South Asia (7.2%)  East-Southeast Asia (16.7%) |
| Urquia et al. 2015 (31) | Australia | Regional | 1999-2008 | Cohort Study | Place of birth | Birth and/or death certificates | All-cause severe maternal morbidity, Uterine rupture | Codes | Medico-administrative data | 636 042 | 138 929 | Western Europe (25.4%)  Eastern Europe (3.4%)  Latin America & the Caribbean (2.9%)  South Asia (12.7%)  East-Southeast Asia (35.3%)  Middle East & North (12.6 %)  Sub-Saharan Africa (7.3%) |
|  | Canada | Regional | 2002-2010 | Cohort Study | Place of birth | Birth and/or death certificates | All-cause severe maternal morbidity, Uterine rupture | Codes | Medico-administrative data | 1 050 688 | 261 107 | Western Europe (6.6%)  Eastern Europe (7.5%)  Latin America & the Caribbean (13.9%)  South Asia (33.5%)  East-Southeast Asia (22.9%)  Middle East & North (8.2 %)  Sub-Saharan Africa (7.5%) |
|  | Denmark | National | 2000-2009 | Cohort Study | Place of birth | Birth and/or death certificates | All-cause severe maternal morbidity, Uterine rupture | Codes | Medico-administrative data | 636 177 | 79 950 | Western Europe (24.8%)  Eastern Europe (6.9%)  Latin America & the Caribbean (2.5%)  South Asia (12.6%)  East-Southeast Asia (10.4%)  Middle East & North (31.4 %)  Sub-Saharan Africa (11.5%) |
| Urquia et al. 2017 (32)  Wanigaratne et al. 2015 (33) | Canada | Regional | 2002-2012 | Cohort Study | Place of birth | Birth and/or death certificates | All-cause severe maternal morbidity, Eclampsia, Uterine rupture, Severe sepsis | Codes | Medico-administrative data | 1 252 543 | 335 544 | Europe and Western nations (15.6%)  South Asia (31.1%)  East Asia, Southeast Asia, and Pacific (22.3%)  Middle East and North Africa (10.0%)  Sub-Saharan Africa (7.3%)  Caribbean (7.9%)  Latin America (5.8%) |
| Wahlberg et al. 2013 (34) | Sweden | National | 1998-2007 | Cohort Study | Place of birth | Birth register | Near-miss, Uterine Rupture, Severe sepsis | Codes | Birth register | 914 474 | 169 344 | Not specified |
| Zanconato et al. 2012 (35) | Italy | Single-center | 2005-2009 | Cohort Study | Place of birth | Medical file | All-cause severe maternal morbidity | Clinical | Medical file | 9395 | 2719 | Not specified |
| Zwart et al. 2008 (36)  Zwart et al. 2010 (37)  Zwart et al. 2011 (38)  Van Hanegem et al. 2011 (39) | Netherlands | National | 2004-2006 | Cohort Study | Place of birth, parents’ place of birth*, and legal status | Birth and/or death certificates | All-cause severe maternal morbidity, Maternal ICU admission, Severe preeclampsia, Severe PPH, Uterine rupture | Clinical | Medical file | 313 564 | Migrant women: 63 132  Asylum seekers : 1310 | Morocco (26.0%)  Turkey (20.5%)  Surinam (14.5%)  Dutch Antilles (6.4%)  Others (32.6%) |

ICU=intensive care unit; PPH=postpartum haemorrhage; NA=not applicable

Papers with data from several countries are presented on separate lines if the risk ratio was calculated for each country

* Some second-generation migrant women might have been included in the migrant women group.

**References**

1. Creanga AA, Berg CJ, Syverson C, Seed K, Bruce FC, Callaghan WM. Race, ethnicity, and nativity differentials in pregnancy-related mortality in the United States: 1993-2006. Obstet Gynecol. 2012;120:261‑8.

2. David M, Razum O, Henrich W, Ramsauer B, Schlembach D, Breckenkamp J. The impact of migration background on maternal near miss. Arch Gynecol Obstet. 2019;300:285‑92.

3. Deneux-Tharaux C, Saucedo M. [Epidemiology of maternal mortality in France, 2010-2012]. Gynecol Obstet Fertil Senol. 2017;45:S8‑21.

4. Diguisto C, Saucedo M, Kallianidis A, Bloemenkamp K, Bødker B, Buoncristiano M, et al. Maternal mortality in eight European countries with enhanced surveillance systems: descriptive population based study. BMJ. 2022;379:e070621.

5. Eslier M, Morello R, Azria E, Dreyfus M. Comparative study of changes in maternal and perinatal morbidity inequalities among migrant and native women over time, between 2008 and 2014 in France. European Journal of Obstetrics & Gynecology and Reproductive Biology. 2020;253:76‑82.

6. Eslier M, Deneux-Tharaux C, Sauvegrain P, Schmitz T, Luton D, Mandelbrot L, et al. Severe maternal morbidity among undocumented migrant women in the PreCARE prospective cohort study. BJOG. 2022;129:1762‑71.

7. Siddiqui A, Deneux-Tharaux C, Luton D, Schmitz T, Mandelbrot L, Estellat C, et al. Maternal obesity and severe pre-eclampsia among immigrant women: a mediation analysis. Sci Rep. 2020;10:5215.

8. Esscher A, Haglund B, Högberg U, Essén B. Excess mortality in women of reproductive age from low-income countries: a Swedish national register study. Eur J Public Health. 2013;23:274‑9.

9. Flood M, Pollock W, McDonald S, Cullinane F, Davey MA. Maternal country of birth and blood transfusion for 370,603 confinements in Victoria. Women and Birth. 2019;32:S14.

10. García-Tizón Larroca S, Arévalo-Serrano J, Ruiz Minaya M, Paya Martinez P, Perez Fernandez Pacheco R, Lizarraga Bonelli S, et al. Maternal mortality trends in Spain during the 2000-2018 period: the role of maternal origin. BMC Public Health. 2022;22:337.

11. Gulersen M, Lenchner E, Grunebaum A, Chervenak FA, Bornstein E. Impact of maternal nativity among maternal racial and ethnic groups at risk for preterm birth. Am J Obstet Gynecol. 2022;226:S725.

12. AIHW: Humphrey MD, Bonello MR, Chughtai A, Macaldowie A, Harris K & Chambers GM 2015. Maternal deaths in Australia 2008–2012. Maternal deaths series no. 5. Cat. no. PER 70. Canberra:AIHW.

13. Australian Institute of Health and Welfare: Humphrey MD et al 2017. Maternal deaths in Australia 2012–2014. Cat. no. PER 92. Canberra: AIHW

14. Australian Institute of Health and Welfare: Humphrey MD et al 2020. Maternal deaths in Australia 2015–2017. Cat. no. PER 106. Canberra: AIHW

15. Jairam JA, Vigod SN, Siddiqi A, Guan J, Boblitz A, Wang X, et al. Severe Maternal Morbidity and Mortality Among Immigrant and Canadian-Born Women Residing Within Low-Income Neighborhoods in Ontario, Canada. JAMA Netw Open. 2023;6:e2256203.

16. AIHW: Johnson S, Bonello MR, Li Z, Hilder L & Sullivan EA. Maternal deaths in Australia 2006– 2010. Maternal deaths series no. 4. Cat. no. PER 61. Canberra: AIHW

17. Kallianidis AF, Schutte JM, Schuringa LEM, Beenakkers ICM, Bloemenkamp KWM, Braams-Lisman BAM, et al. Confidential enquiry into maternal deaths in the Netherlands, 2006-2018. Acta Obstet Gynecol Scand. 2022;101:441‑9.

18. Knight M, Tuffnell D, Kenyon S, Shakespeare J, Gray R, Kurinczuk JJ (Eds.) on behalf of MBRRACE-UK. Saving Lives, Improving Mothers’ Care - Surveillance of maternal deaths in the UK 2011-13 and lessons learned to inform maternity care from the UK and Ireland Confidential Enquiries into Maternal Deaths and Morbidity 2009-13. Oxford: National Perinatal Epidemiology Unit, University of Oxford 2015.

19. Knight M, Nair M, Tuffnell D, Shakespeare J, Kenyon S, Kurinczuk JJ (Eds.) on behalf of MBRRACE-UK. Saving Lives, Improving Mothers’ Care - Lessons learned to inform maternity care from the UK and Ireland Confidential Enquiries into Maternal Deaths and Morbidity 2013–15. Oxford: National Perinatal Epidemiology Unit, University of Oxford 2017.

20. Knight M, Bunch K, Tuffnell D, Shakespeare J, Kotnis R, Kenyon S, Kurinczuk JJ (Eds.) on behalf of MBRRACE-UK. Saving Lives, Improving Mothers’ Care - Lessons learned to inform maternity care from the UK and Ireland Confidential Enquiries into Maternal Deaths and Morbidity 2016-18. Oxford: National Perinatal Epidemiology Unit, University of Oxford 2020.

21. Leonard SA, Main EK, Lyell DJ, Carmichael SL, Kennedy CJ, Johnson C, et al. Obstetric comorbidity scores and disparities in severe maternal morbidity across marginalized groups. Am J Obstet Gynecol MFM. 2022;4:100530.

22. Medcalf KE, Park AL, Vermeulen MJ, Ray JG. Maternal Origin and Risk of Neonatal and Maternal ICU Admission*: Critical Care Medicine. 2016;44:1314‑26.

23. Mujahid MS, Kan P, Leonard SA, Hailu EM, Wall-Wieler E, Abrams B, et al. Birth hospital and racial and ethnic differences in severe maternal morbidity in the state of California. American Journal of Obstetrics and Gynecology. 2021;224:219.e1-219.e15.

24. Wall-Wieler E, Bane S, Lee HC, Carmichael SL. Severe maternal morbidity among U.S.- and foreign-born Asian and Pacific Islander women in California. Annals of Epidemiology. 2020;52:60-63.e2.

25. Reime B, Janssen PA, Farris L, Borde T, Hellmers C, Myezwa H, et al. Maternal near-miss among women with a migrant background in Germany. Acta Obstet Gynecol Scand. 2012;91:824‑9.

26. Saucedo M, Deneux-Tharaux C, Pour le Comité National d’Experts sur la Mortalité Maternelle. [Maternal Mortality, Frequency, causes, women’s profile and preventability of deaths in France, 2013-2015]. Gynecol Obstet Fertil Senol. 2021;49:9‑26.

27. Schutte JM, Steegers E a. P, Schuitemaker NWE, Santema JG, Boer K de, Pel M, et al. Rise in maternal mortality in the Netherlands. BJOG: An International Journal of Obstetrics & Gynaecology. 2010;117:399‑406.

28. Singh GK. Trends and Social Inequalities in Maternal Mortality in the United States, 1969-2018. Int J MCH AIDS. 2020;10:29‑42.

29. Turner JM, Spink K, Fox J, Kumar SS. Refugee women have significantly poorer perinatal outcomes despite a specialised refugee midwifery service. J Paediatr Child Health. 2020;56:128‑9.

30. Urquia M, Glazier R, Gagnon A, Mortensen L, Nybo Andersen AM, Janevic T, et al. Disparities in pre-eclampsia and eclampsia among immigrant women giving birth in six industrialised countries. BJOG: An International Journal of Obstetrics & Gynaecology. 2014;121:1492‑500.

31. Urquia ML, Glazier RH, Mortensen L, Nybo-Andersen AM, Small R, Davey MA, et al. Severe maternal morbidity associated with maternal birthplace in three high-immigration settings. The European Journal of Public Health. 2015;25:620‑5.

32. Urquia ML, Wanigaratne S, Ray JG, Joseph KS. Severe Maternal Morbidity Associated With Maternal Birthplace: A Population-Based Register Study. Journal of Obstetrics and Gynaecology Canada. 2017;39:978‑87.

33. Wanigaratne S, Cole DC, Bassil K, Hyman I, Moineddin R, Urquia ML. Contribution of HIV to Maternal Morbidity Among Refugee Women in Canada. Am J Public Health. 2015;105:2449‑56.

34. Wahlberg Å, Rööst M, Haglund B, Högberg U, Essén B. Increased risk of severe maternal morbidity (near-miss) among immigrant women in Sweden: a population register-based study. BJOG: Int J Obstet Gy. 2013;120:1605‑12.

35. Zanconato G, Cavaliere E, Iacovella C, Vassanelli A, Schweiger V, Cipriani S, et al. Severe maternal morbidity in a tertiary care centre of northern Italy: a 5-year review. The Journal of Maternal-Fetal & Neonatal Medicine. 2012;25:1025‑8.

36. Zwart J, Richters J, Öry F, de Vries J, Bloemenkamp K, van Roosmalen J. Severe maternal morbidity during pregnancy, delivery and puerperium in the Netherlands: a nationwide population-based study of 371 000 pregnancies. BJOG: An International Journal of Obstetrics & Gynaecology. 2008;115:842‑50.

37. Zwart JJ, Dupuis JRO, Richters A, Ory F, van Roosmalen J. Obstetric intensive care unit admission: a 2-year nationwide population-based cohort study. Intensive Care Med. 2010;36:256‑63.

38. Zwart JJ, Jonkers MD, Richters A, Öry F, Bloemenkamp KW, Duvekot JJ, et al. Ethnic disparity in severe acute maternal morbidity: a nationwide cohort study in the Netherlands. European Journal of Public Health. 2011;21:229‑34.

39. Van Hanegem N, Miltenburg AS, Zwart JJ, Bloemenkamp KWM, Van Roosmalen J. Severe acute maternal morbidity in asylum seekers: a two-year nationwide cohort study in the Netherlands: Severe maternal morbidity in asylum seekers. Acta Obstetricia et Gynecologica Scandinavica. 2011;90:1010‑6.
