# Supplementary material for: Association between migration and severe maternal outcomes in high-income countries: Systematic review and meta-analysis
Source: PLoS Med. 2023 Jun 22;20(6):e1004257. doi: 10.1371/journal.pmed.1004257 (PMC10328365; doi:10.1371/journal.pmed.1004257)
Supplement: S6 Table — (DOCX) [file pmed.1004257.s006.docx]

S6 Table. Methodological quality of the studies included (listed in alphabetical order) assessed with the Newcastle-Ottawa Scale (NOS)

| **Author** | **Selection** | | | | **Comparability** | **Outcome** | | | **Total (max score: 9⁎)** |
| --- | --- | --- | --- | --- | --- | --- | --- | --- | --- |
|  | **Represent-ativeness of exposed cohort (max score: ⁎)** | **Selection of non-exposed (max score: ⁎)** | **Ascertain-ment of exposure (max score: ⁎)** | **Demonstration that outcome of interest was not present at start of study (max score: ⁎)** | **Comparability of cohorts on the basis of design or analysis (max score: ⁑)** | **Assessment of outcome (max score: ⁎)** | **Was follow-up long enough for outcomes to occur (max score: ⁎)** | **Adequacy of follow up (max score: ⁎)** |  |
| Creanga et al. 2012 (1) | ⁎ | ⁎ | ⁎ | ⁎ | - | ⁎ | ⁎ | ⁎ | 7 |
| David et al. 2019 (2) | - | ⁎ | ⁎ | ⁎ | - | ⁎ | ⁎ | ⁎ | 6 |
| Deneux-Tharaux et al. 2017 (3) | ⁎ | ⁎ | ⁎ | ⁎ | - | ⁎ | ⁎ | ⁎ | 7 |
| Diguisto et al. 2022 (4) | ⁎ | ⁎ | ⁎ | ⁎ | - | ⁎ | ⁎ | ⁎ | 7 |
| Eslier et al. 2020 (5) | ⁎ | ⁎ | ⁎ | ⁎ | - | ⁎ | ⁎ | ⁎ | 7 |
| Eslier et al. 2022 (6) | ⁎ | ⁎ | ⁎ | ⁎ | ⁎ | ⁎ | ⁎ | ⁎ | 8 |
| Esscher et al. 2013 (7) | ⁎ | ⁎ | ⁎ | ⁎ | - | ⁎ | ⁎ | ⁎ | 7 |
| Flood et al. 2020 (8) | ⁎ | ⁎ | ⁎ | ⁎ | ⁎ | ⁎ | ⁎ | ⁎ | 8 |
| García‑Tizón Larroca et al. 2022 (9) | ⁎ | ⁎ | ⁎ | ⁎ | ⁎ | ⁎ | ⁎ | - | 8 |
| Gulersen et al. 2022 (10) | ⁎ | ⁎ | ⁎ | ⁎ | ⁎ | ⁎ | ⁎ | - | 7 |
| Humphrey et al. 2015 (11) | ⁎ | ⁎ | ⁎ | ⁎ | - | ⁎ | ⁎ | ⁎ | 7 |
| Humphrey et al. 2017 (12) | ⁎ | ⁎ | ⁎ | ⁎ | - | ⁎ | ⁎ | ⁎ | 7 |
| Humphrey et al. 2020 (13) | ⁎ | ⁎ | ⁎ | ⁎ | - | ⁎ | ⁎ | ⁎ | 7 |
| Jairam et al. 2023 (14) | ⁎ | ⁎ | ⁎ | ⁎ | ⁎ | ⁎ | ⁎ | ⁎ | 8 |
| Johnson et al. 2014 (15) | ⁎ | ⁎ | ⁎ | ⁎ | - | ⁎ | ⁎ | ⁎ | 7 |
| Kallianidis et al. 2022 (16) | ⁎ | ⁎ | ⁎ | ⁎ | - | ⁎ | ⁎ | ⁎ | 7 |
| Knight et al. 2015 (17) | ⁎ | ⁎ | ⁎ | ⁎ | - | ⁎ | ⁎ | ⁎ | 7 |
| Knight et al. 2017 (18) | ⁎ | ⁎ | ⁎ | ⁎ | - | ⁎ | ⁎ | ⁎ | 7 |
| Knight et al. 2020 (19) | ⁎ | ⁎ | ⁎ | ⁎ | - | ⁎ | ⁎ | ⁎ | 7 |
| Leonard et al. 2021 (20) | ⁎ | ⁎ | ⁎ | ⁎ | ⁎ | ⁎ | ⁎ | - | 7 |
| Medcalf et al. 2016 (21) | ⁎ | ⁎ | ⁎ | ⁎ | ⁎ | ⁎ | ⁎ | ⁎ | 8 |
| Mujahid et al. 2020 (22) | ⁎ | ⁎ | ⁎ | ⁎ | - | ⁎ | ⁎ | ⁎ | 7 |
| Reime et al. 2012 (23) | ⁎ | ⁎ | ⁎ | ⁎ | ⁎ | ⁎ | ⁎ | - | 7 |
| Saucedo et al. 2021 (24) | ⁎ | ⁎ | ⁎ | ⁎ | - | ⁎ | ⁎ | ⁎ | 7 |
| Schutte et al. 2009 (25) | ⁎ | ⁎ | ⁎ | ⁎ | - | ⁎ | ⁎ | - | 6 |
| Singh et al. 2021 (26) | ⁎ | ⁎ | ⁎ | ⁎ | - | ⁎ | ⁎ | ⁎ | 7 |
| Siddiqui et al. 2020 (27) | ⁎ | ⁎ | ⁎ | ⁎ | ⁎ | ⁎ | ⁎ | ⁎ | 8 |
| Turner et al. 2020 (28) | ⁎ | ⁎ | ⁎ | ⁎ | - | ⁎ | ⁎ | - | 6 |
| Urquia et al. 2014(29) | ⁎ | ⁎ | ⁎ | ⁎ | ⁎ | ⁎ | ⁎ | ⁎ | 8 |
| Urquia et al. 2015 (30) | ⁎ | ⁎ | ⁎ | ⁎ | ⁎ | ⁎ | ⁎ | - | 7 |
| Urquia et al. 2017 (31) | ⁎ | ⁎ | ⁎ | ⁎ | ⁎ | ⁎ | ⁎ | ⁎ | 8 |
| Van Hanegem et al. 2011 (32) | ⁎ | ⁎ | ⁎ | ⁎ | - | ⁎ | ⁎ | ⁎ | 7 |
| Wahlberg et al. 2013 (33) | ⁎ | ⁎ | ⁎ | ⁎ | ⁎ | ⁎ | ⁎ | ⁎ | 8 |
| Wall-Wieler et al. 2020 (34) | ⁎ | ⁎ | ⁎ | ⁎ | - | ⁎ | ⁎ | ⁎ | 7 |
| Wanigaratne et al. 2015 (35) | ⁎ | ⁎ | ⁎ | ⁎ | ⁎ | ⁎ | ⁎ | - | 7 |
| Zanconato et al. 2012 (36) | ⁎ | ⁎ | ⁎ | ⁎ | ⁎ | ⁎ | ⁎ | - | 7 |
| Zwart et al. 2008 (37) | ⁎ | ⁎ | ⁎ | ⁎ | - | ⁎ | ⁎ | ⁎ | 7 |
| Zwart et al. 2010 (38) | ⁎ | ⁎ | ⁎ | ⁎ | - | ⁎ | ⁎ | ⁎ | 7 |
| Zwart et al. 2011 (39) | ⁎ | ⁎ | ⁎ | ⁎ | - | ⁎ | ⁎ | ⁎ | 7 |

**References**

1. Creanga AA, Berg CJ, Syverson C, Seed K, Bruce FC, Callaghan WM. Race, ethnicity, and nativity differentials in pregnancy-related mortality in the United States: 1993-2006. Obstet Gynecol. 2012;120:261‑8.

2. David M, Razum O, Henrich W, Ramsauer B, Schlembach D, Breckenkamp J. The impact of migration background on maternal near miss. Arch Gynecol Obstet. 2019;300:285‑92.

3. Deneux-Tharaux C, Saucedo M. [Epidemiology of maternal mortality in France, 2010-2012]. Gynecol Obstet Fertil Senol. 2017;45:S8‑21.

4. Diguisto C, Saucedo M, Kallianidis A, Bloemenkamp K, Bødker B, Buoncristiano M, et al. Maternal mortality in eight European countries with enhanced surveillance systems: descriptive population based study. BMJ. 2022;379:e070621.

5. Eslier M, Morello R, Azria E, Dreyfus M. Comparative study of changes in maternal and perinatal morbidity inequalities among migrant and native women over time, between 2008 and 2014 in France. European Journal of Obstetrics & Gynecology and Reproductive Biology. 2020;253:76‑82.

6. Eslier M, Deneux-Tharaux C, Sauvegrain P, Schmitz T, Luton D, Mandelbrot L, et al. Severe maternal morbidity among undocumented migrant women in the PreCARE prospective cohort study. BJOG. 2022;129:1762‑71.

7. Esscher A, Haglund B, Högberg U, Essén B. Excess mortality in women of reproductive age from low-income countries: a Swedish national register study. Eur J Public Health. 2013;23:274‑9.

8. Flood M, Pollock W, McDonald S, Cullinane F, Davey MA. Maternal country of birth and blood transfusion for 370,603 confinements in Victoria. Women and Birth. 2019;32:S14.

9. García-Tizón Larroca S, Arévalo-Serrano J, Ruiz Minaya M, Paya Martinez P, Perez Fernandez Pacheco R, Lizarraga Bonelli S, et al. Maternal mortality trends in Spain during the 2000-2018 period: the role of maternal origin. BMC Public Health. 2022;22:337.

10. Gulersen M, Lenchner E, Grunebaum A, Chervenak FA, Bornstein E. Impact of maternal nativity among maternal racial and ethnic groups at risk for preterm birth. Am J Obstet Gynecol. 2022;226:S725.

11. AIHW: Humphrey MD, Bonello MR, Chughtai A, Macaldowie A, Harris K & Chambers GM 2015. Maternal deaths in Australia 2008–2012. Maternal deaths series no. 5. Cat. no. PER 70. Canberra:AIHW.

12. Australian Institute of Health and Welfare: Humphrey MD et al 2017. Maternal deaths in Australia 2012–2014. Cat. no. PER 92. Canberra: AIHW

13. Australian Institute of Health and Welfare: Humphrey MD et al 2020. Maternal deaths in Australia 2015–2017. Cat. no. PER 106. Canberra: AIHW

14. Jairam JA, Vigod SN, Siddiqi A, Guan J, Boblitz A, Wang X, et al. Severe Maternal Morbidity and Mortality Among Immigrant and Canadian-Born Women Residing Within Low-Income Neighborhoods in Ontario, Canada. JAMA Netw Open. 2023;6:e2256203.

15. AIHW: Johnson S, Bonello MR, Li Z, Hilder L & Sullivan EA. Maternal deaths in Australia 2006– 2010. Maternal deaths series no. 4. Cat. no. PER 61. Canberra: AIHW

16. Kallianidis AF, Schutte JM, Schuringa LEM, Beenakkers ICM, Bloemenkamp KWM, Braams-Lisman BAM, et al. Confidential enquiry into maternal deaths in the Netherlands, 2006-2018. Acta Obstet Gynecol Scand. 2022;101:441‑9.

17. Knight M, Tuffnell D, Kenyon S, Shakespeare J, Gray R, Kurinczuk JJ (Eds.) on behalf of MBRRACE-UK. Saving Lives, Improving Mothers’ Care - Surveillance of maternal deaths in the UK 2011-13 and lessons learned to inform maternity care from the UK and Ireland Confidential Enquiries into Maternal Deaths and Morbidity 2009-13. Oxford: National Perinatal Epidemiology Unit, University of Oxford 2015.

18. Knight M, Nair M, Tuffnell D, Shakespeare J, Kenyon S, Kurinczuk JJ (Eds.) on behalf of MBRRACE-UK. Saving Lives, Improving Mothers’ Care - Lessons learned to inform maternity care from the UK and Ireland Confidential Enquiries into Maternal Deaths and Morbidity 2013–15. Oxford: National Perinatal Epidemiology Unit, University of Oxford 2017.

19. Knight M, Bunch K, Tuffnell D, Shakespeare J, Kotnis R, Kenyon S, Kurinczuk JJ (Eds.) on behalf of MBRRACE-UK. Saving Lives, Improving Mothers’ Care - Lessons learned to inform maternity care from the UK and Ireland Confidential Enquiries into Maternal Deaths and Morbidity 2016-18. Oxford: National Perinatal Epidemiology Unit, University of Oxford 2020.

20. Leonard SA, Main EK, Lyell DJ, Carmichael SL, Kennedy CJ, Johnson C, et al. Obstetric comorbidity scores and disparities in severe maternal morbidity across marginalized groups. Am J Obstet Gynecol MFM. 2022;4:100530.

21. Medcalf KE, Park AL, Vermeulen MJ, Ray JG. Maternal Origin and Risk of Neonatal and Maternal ICU Admission*: Critical Care Medicine. 2016;44:1314‑26.

22. Mujahid MS, Kan P, Leonard SA, Hailu EM, Wall-Wieler E, Abrams B, et al. Birth hospital and racial and ethnic differences in severe maternal morbidity in the state of California. American Journal of Obstetrics and Gynecology. 2021;224:219.e1-219.e15.

23. Reime B, Janssen PA, Farris L, Borde T, Hellmers C, Myezwa H, et al. Maternal near-miss among women with a migrant background in Germany. Acta Obstet Gynecol Scand. 2012;91:824‑9.

24. Saucedo M, Deneux-Tharaux C, Pour le Comité National d’Experts sur la Mortalité Maternelle. [Maternal Mortality, Frequency, causes, women’s profile and preventability of deaths in France, 2013-2015]. Gynecol Obstet Fertil Senol. 2021;49:9‑26.

25. Schutte JM, Steegers E a. P, Schuitemaker NWE, Santema JG, Boer K de, Pel M, et al. Rise in maternal mortality in the Netherlands. BJOG: An International Journal of Obstetrics & Gynaecology. 2010;117:399‑406.

26. Singh GK. Trends and Social Inequalities in Maternal Mortality in the United States, 1969-2018. Int J MCH AIDS. 2020;10:29‑42.

27. Siddiqui A, Deneux-Tharaux C, Luton D, Schmitz T, Mandelbrot L, Estellat C, et al. Maternal obesity and severe pre-eclampsia among immigrant women: a mediation analysis. Sci Rep. 2020;10:5215.

28. Turner JM, Spink K, Fox J, Kumar SS. Refugee women have significantly poorer perinatal outcomes despite a specialised refugee midwifery service. J Paediatr Child Health. 2020;56:128‑9.

29. Urquia M, Glazier R, Gagnon A, Mortensen L, Nybo Andersen AM, Janevic T, et al. Disparities in pre-eclampsia and eclampsia among immigrant women giving birth in six industrialised countries. BJOG: An International Journal of Obstetrics & Gynaecology. 2014;121:1492‑500.

30. Urquia ML, Glazier RH, Mortensen L, Nybo-Andersen AM, Small R, Davey MA, et al. Severe maternal morbidity associated with maternal birthplace in three high-immigration settings. The European Journal of Public Health. 2015;25:620‑5.

31. Urquia ML, Wanigaratne S, Ray JG, Joseph KS. Severe Maternal Morbidity Associated With Maternal Birthplace: A Population-Based Register Study. Journal of Obstetrics and Gynaecology Canada. 2017;39:978‑87.

32. Van Hanegem N, Miltenburg AS, Zwart JJ, Bloemenkamp KWM, Van Roosmalen J. Severe acute maternal morbidity in asylum seekers: a two-year nationwide cohort study in the Netherlands: Severe maternal morbidity in asylum seekers. Acta Obstetricia et Gynecologica Scandinavica. 2011;90:1010‑6.

33. Wahlberg Å, Rööst M, Haglund B, Högberg U, Essén B. Increased risk of severe maternal morbidity (near-miss) among immigrant women in Sweden: a population register-based study. BJOG: Int J Obstet Gy. 2013;120:1605‑12.

34. Wall-Wieler E, Bane S, Lee HC, Carmichael SL. Severe maternal morbidity among U.S.- and foreign-born Asian and Pacific Islander women in California. Annals of Epidemiology. 2020;52:60-63.e2.

35. Wanigaratne S, Cole DC, Bassil K, Hyman I, Moineddin R, Urquia ML. The influence of refugee status and secondary migration on preterm birth. J Epidemiol Community Health. 2016;70:622‑8.

36. Zanconato G, Cavaliere E, Iacovella C, Vassanelli A, Schweiger V, Cipriani S, et al. Severe maternal morbidity in a tertiary care centre of northern Italy: a 5-year review. The Journal of Maternal-Fetal & Neonatal Medicine. 2012;25:1025‑8.

37. Zwart J, Richters J, Öry F, de Vries J, Bloemenkamp K, van Roosmalen J. Severe maternal morbidity during pregnancy, delivery and puerperium in the Netherlands: a nationwide population-based study of 371 000 pregnancies. BJOG: An International Journal of Obstetrics & Gynaecology. 2008;115:842‑50.

38. Zwart JJ, Dupuis JRO, Richters A, Ory F, van Roosmalen J. Obstetric intensive care unit admission: a 2-year nationwide population-based cohort study. Intensive Care Med. 2010;36:256‑63.

39. Zwart JJ, Jonkers MD, Richters A, Öry F, Bloemenkamp KW, Duvekot JJ, et al. Ethnic disparity in severe acute maternal morbidity: a nationwide cohort study in the Netherlands. European Journal of Public Health. 2011;21:229‑34.
