# Supplementary material for: Association between migration and severe maternal outcomes in high-income countries: Systematic review and meta-analysis
Source: PLoS Med. 2023 Jun 22;20(6):e1004257. doi: 10.1371/journal.pmed.1004257 (PMC10328365; doi:10.1371/journal.pmed.1004257)
Supplement: S7 Table — (DOCX) [file pmed.1004257.s007.docx]

S7 Table. Amendments made to the protocol PROSPERO

| **Initial protocol** | **Actual Methods section** |
| --- | --- |
| We will include mental health in outcomes. | We excluded studies about mental health, because we thought that it was more relevant to study this large topic in another systematic review. |
| We will include observational studies (including case-control studies, cross-sectional studies and cohort studies (including exposed-non-exposed studies)), systematic reviews and meta-analysis | Case-control studies were excluded, because this type of study could not estimate relevant risk ratio between migrant and native women. |
| We will restrict our search to studies in English, French and Greek (because one reviewer is Greek) | We thought it was more relevant to search without language restriction. |
