# Supplementary material for: Association between migration and severe maternal outcomes in high-income countries: Systematic review and meta-analysis
Source: PLoS Med. 2023 Jun 22;20(6):e1004257. doi: 10.1371/journal.pmed.1004257 (PMC10328365; doi:10.1371/journal.pmed.1004257)
Supplement: S2 Fig — (DOCX) [file pmed.1004257.s009.docx]

S2 Figure. All-cause severe maternal morbidity in migrant women and native-born women


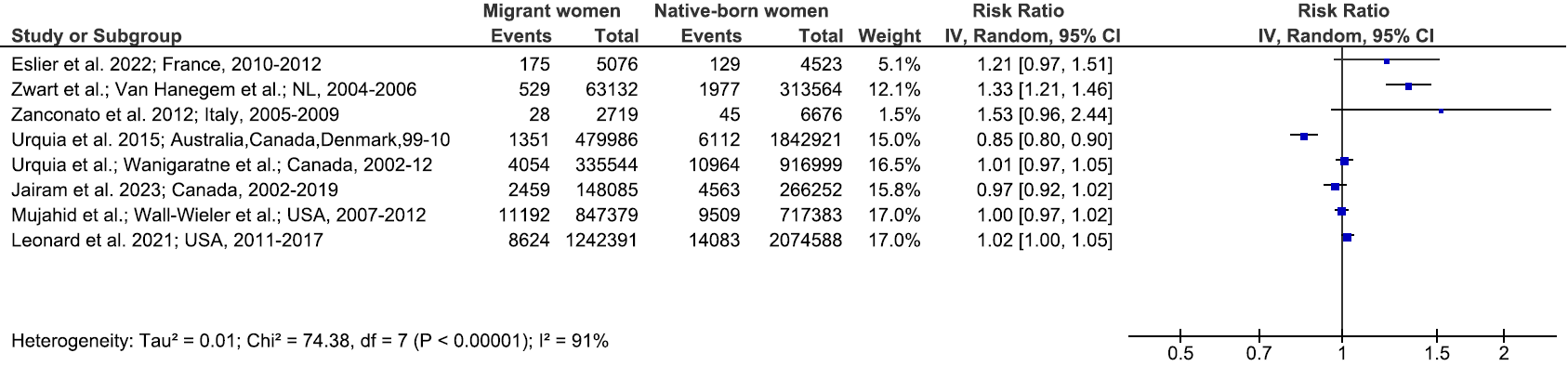


CI=confidence interval; IV=inverse variance; df=degrees of freedom

The squares show the unadjusted risk ratios (RR) with their 95% confidence intervals. The size of the squares indicates the weight of the study. The confidence interval is shown with lines.
