# Supplementary material for: Association between migration and severe maternal outcomes in high-income countries: Systematic review and meta-analysis
Source: PLoS Med. 2023 Jun 22;20(6):e1004257. doi: 10.1371/journal.pmed.1004257 (PMC10328365; doi:10.1371/journal.pmed.1004257)
Supplement: S3 Fig — (DOCX) [file pmed.1004257.s010.docx]

S3 Figure. Maternal intensive care unit admission in migrant women and native-born women, stratified by host country


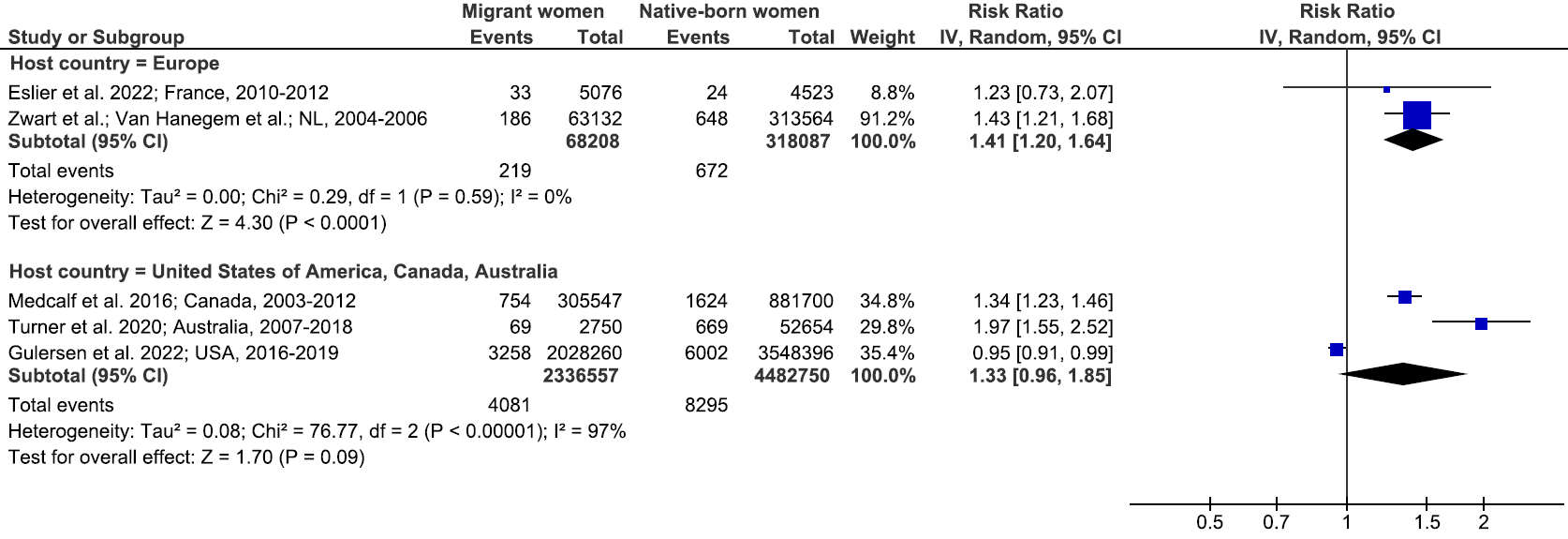


CI=confidence interval; IV=inverse variance; df=degrees of freedom

Unadjusted risk ratios are random-effects estimates calculated by the DerSimonian and Laird method. The data markers show the unadjusted risk ratios (RR) with their 95% confidence intervals. The size of the data markers indicates the weight of the study. Diamonds show the pooled unadjusted risk ratios. The confidence interval is shown with lines.
