# Supplementary material for: Association between migration and severe maternal outcomes in high-income countries: Systematic review and meta-analysis
Source: PLoS Med. 2023 Jun 22;20(6):e1004257. doi: 10.1371/journal.pmed.1004257 (PMC10328365; doi:10.1371/journal.pmed.1004257)
Supplement: S5 Fig — (DOCX) [file pmed.1004257.s012.docx]

S5 Figure. Funnel plot for studies reporting maternal mortality overall


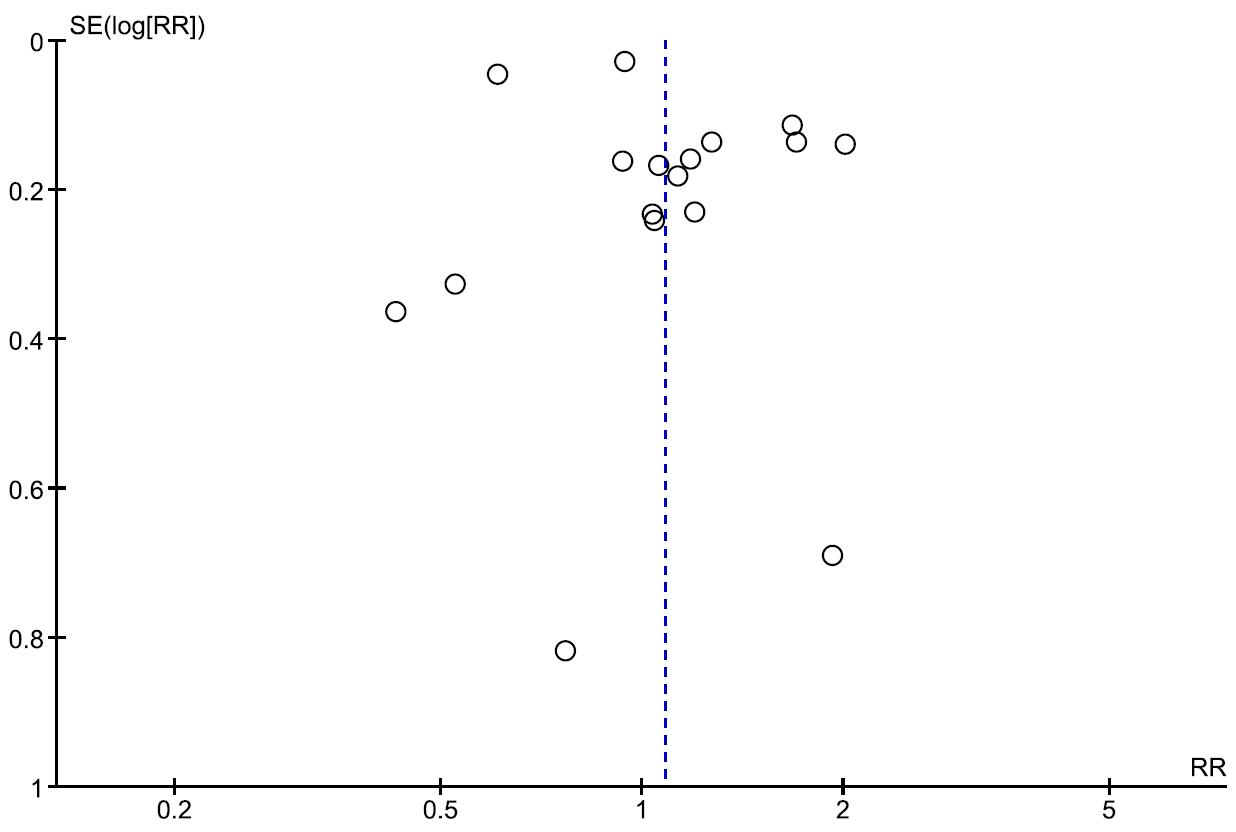


SE = Standard Error; RR=Risk Ratio

The x-axis represents the unadjusted risk ratio between migrant and native-born women; the y-axis represents the standard error of the log risk ratio. Each plotted point represents a study. The vertical line represents the pooled risk ratio found in the meta-analysis.
