# Supplementary material for: Association between migration and severe maternal outcomes in high-income countries: Systematic review and meta-analysis
Source: PLoS Med. 2023 Jun 22;20(6):e1004257. doi: 10.1371/journal.pmed.1004257 (PMC10328365; doi:10.1371/journal.pmed.1004257)
Supplement: Attachment — Submitted filename: eslier.pdf [file pmed.1004257.s014.pdf]

This paper reviews studies of the relationship between place of birth and maternal health outcomes in women who have migrated.

I was asked for a statistical report and I interpret that to include all aspects of the design and conduct of the study.

Points of detail

Page 5 If any of the articles were in languages other than English then somewhere we need to know how they were translated or otherwise dealt with.

Page 6 If authors were asked to supply extra data then I think that (a) we should be told which items we are not going to be able to find in the original publications (b) the people who supplied it should be acknowledged. If this is what the list on page 15 is then perhaps state there why they are being acknowledged. That would address point (b) but not (a).

Page 6 How did the authors score studies in which they had been involved? I assume that the Eslier who is an author on some of the references is the same as the current first author. Since quality scores do not play a major part subsequently it would be acceptable not to score those studies. Finding someone else to do it who does not have a conflict of interest would be almost impossible.

Page 7 Confidence intervals for I^2 would also be helpful to reveal our uncertainty about the heterogeneity. I know we did not use to use them but I am persuaded by Ioannidis et al. (2007) that we should. For instance in Figure 2A for the first analysis $I^2 = 68$ (32 to 85) which is quite imprecise although the second one is more precise 93 (88 to 96).

Page 7 I do not quite understand the point about heterogeneity here. Do the authors mean that they decided on scientific grounds that there would be heterogeneity and so decided to use random effect models (which seems to me the way to go) or did they do it dependent on observed statistical heterogeneity? It looks like the former but it was not clear to me.

Page 8 Providing a list of excluded studies is helpful but seldom done.

Page 8 and elsewhere I wonder whether databases is the best word here? It surprised me on first reading. Would studies or datasets be acceptable alternatives?

Page 9 What does vital statistics mean here? For me it means the central records kept of births and deaths and the publications which national statistics agencies produce from them but that does not seem relevant here.

Page 11 I think what Figures 5a and 5b show is the limited value of funnel plots when there is high heterogeneity (as the authors themselves suggest). The plots do not present any concerns.

Table S5 Some of the entries in the last column are not migrants place of birth as stated in the heading. Specifically Creaga has ethnicity not place of birth, Esscher has income classification not region as does Wahlberg.

Table S5 Are the studies which have not specified as place of birth really capable of being included in this review?

Table S5 In the first two Knight studies the most numerous group was women born in Poland (which did not surprise me) but in the third study they do not seem to appear at all. Is this really the case?

Points of more substance

Multi–level nature of studies

The individual studies represented in the data consist of several sets which clearly come from the same group, using similar methods, and studying similar groups of women. Since these are not independent estimates one of the assumptions underlying meta–analysis, independence of the primary studies, seems hard to justify. There are various approaches to dealing with this like multi–level meta–analysis, aggregation of studies from the same source. At a minimum the problem needs noting and discussing.

Host country and region are not independent

Differences found between host countries are confounded by differences between the regions from which the women migrated. I would suggest that we need a meta–regression of all the studies with host country and birth region as moderators to attempt to separate the effects. This would also give us an estimate of the differences between levels which presenting by subset does not do.

Summary

Points for clarification and some queries about the appropriateness of the analysis choices.

Michael Dewey

References

J Ioannidis, N Patsopoulos, and E Evangelou. Uncertainty in heterogeneity estimates in meta–analyses. *British Medical Journal*, 335:914–916, 2007.
