# Supplementary material for: Association between migration and severe maternal outcomes in high-income countries: Systematic review and meta-analysis
Source: PLoS Med. 2023 Jun 22;20(6):e1004257. doi: 10.1371/journal.pmed.1004257 (PMC10328365; doi:10.1371/journal.pmed.1004257)
Supplement: Attachment — Submitted filename: Eslier_Answers to reviewers_R2.docx [file pmed.1004257.s015.docx]

Manuscript number: PMEDICINE-D-22-03069R1

Title: Association between migration and severe maternal outcomes in high income countries: systematic review and meta-analysis

Responses to reviewers

**Answers to Philippa Dodd**

1- We request that you upload any figures associated with your paper as individual TIF or EPS files with 300dpi resolution at resubmission; please read our figure guidelines for more information on our requirements: http://journals.plos.org/plosmedicine/s/figures. While revising your submission, please upload your figure files to the PACE digital diagnostic tool, https://pacev2.apexcovantage.com/. PACE helps ensure that figures meet PLOS requirements. To use PACE, you must first register as a user. Then, login and navigate to the UPLOAD tab, where you will find detailed instructions on how to use the tool. If you encounter any issues or have any questions when using PACE, please email us at PLOSMedicine@plos.org.

- **Response:**

**We have uploaded all figures associated with our paper as individual TIF files with 300dpi resolution according to your figure guidelines. We have checked all figures with the PACE digital diagnostic tool.**

2- We ask every co-author listed on the manuscript to fill in a contributing author statement, making sure to declare all competing interests. If any of the co-authors have not filled in the statement, we will remind them to do so when the paper is revised. If all statements are not completed in a timely fashion this could hold up the re-review process. If new competing interests are declared later in the revision process, this may also hold up the submission. Should there be a problem getting one of your co-authors to fill in a statement we will be in contact. YOU MUST NOT ADD OR REMOVE AUTHORS UNLESS YOU HAVE ALERTED THE EDITOR HANDLING THE MANUSCRIPT TO THE CHANGE AND THEY SPECIFICALLY HAVE AGREED TO IT. You can see our competing interests policy here: http://journals.plos.org/plosmedicine/s/competing-interests.

- **Response:**

**All co-authors listed on the manuscript have completed a contributing author statement to declare all competing interests. We have uploaded all authors’ statements.**

3- To enhance the reproducibility of your results, we recommend that you deposit your laboratory protocols in protocols.io, where a protocol can be assigned its own identifier (DOI) such that it can be cited independently in the future. Additionally, PLOS ONE offers an option to publish peer-reviewed clinical study protocols. Read more information on sharing protocols at https://plos.org/protocols?utm_medium=editorial-email&utm_source=authorletters&utm_campaign=protocols

- **Response:**

**The review protocol was already registered with the PROSPERO International Prospective Register of Systematic Reviews, on its website. The web link to the published protocol is https://www.crd.york.ac.uk/PROSPEROFILES/224193_PROTOCOL_20210824.pdf**

4- Please ensure that the paper adheres to the PLOS Data Availability Policy (see http://journals.plos.org/plosmedicine/s/data-availability), which requires that all data underlying the study's findings be provided in a repository or as Supporting Information. For data residing with a third party, authors are required to provide instructions with contact information for obtaining the data. PLOS journals do not allow statements supported by "data not shown" or "unpublished results." For such statements, authors must provide supporting data or cite public sources that include it.

- **Response:**

**We confirm that all data underlying the findings described in the manuscript are available in the main and supporting information.**

**Answers to the Editors**

## **General**

1. Please revise your use of language when referring to both ‘migrants’ and ‘natives’, suggest instead native-born women and migrant women.

- **Response:**

**We agree with this suggestion and have revised the whole manuscript, figures and supplementary material accordingly.**

2. Please check carefully for the use of appropriate grammar, a number of minor errors negatively impacting reader accessibility were identified, including by the reviewers. We suggest inviting a native English speaker to proof read the manuscript prior to re-submission. Should the manuscript proceed successfully through the peer review process to publication, our copyeditors can help you further.

- **Response:**

**This manuscript had already been edited by a native-English-speaking professional biomedical editor and translator, who has again reviewed and edited extensively before being resubmitted.**

3. Please update your search to the present time. PLOS Medicine requires that all SR/MAs are updated to within 6 months of an anticipated publication date.

- **Response:**

**We updated our search on April 18, 2023 and have changed the search dates in the manuscript, figures and supplementary material accordingly. This update yielded 3 additional studies that are now included in our manuscript:**

1. **Diguisto C, Saucedo M, Kallianidis A, Bloemenkamp K, Bødker B, Buoncristiano M, et al. Maternal mortality in eight European countries with enhanced surveillance systems: descriptive population based study. BMJ. 2022;e070621.**
2. **Jairam JA, Vigod SN, Siddiqi A, Guan J, Boblitz A, Wang X, et al. Severe Maternal Morbidity and Mortality Among Immigrant and Canadian-Born Women Residing Within Low-Income Neighborhoods in Ontario, Canada. JAMA Netw Open. 2023;6:e2256203.**
3. **Kallianidis AF, Schutte JM, Schuringa LEM, Beenakkers ICM, Bloemenkamp KWM, Braams-Lisman BAM, et al. Confidential enquiry into maternal deaths in the Netherlands, 2006-2018. Acta Obstet Gynecol Scand. 2022;101:441‑9.**

**Data from these 3 papers have been included in our meta-analysis when appropriate, and forest plots and pooled estimates have been updated accordingly.**

## **Comments from the academic editor**

4. I agree that this is an important area. However, as presented, the findings do not add a huge amount to the literature. In the main this is due to the simplistic comparison of migrant and native, as pointed out by Reviewer 4.

It seems appropriate to challenge the authors to provide a major revision, including reanalyses as suggested by the statistical reviewer, and more nuanced discussion of the limitations as suggested by the other reviewers.

- **Response:**

**Thank you for your comments. As requested, our analysis has been completed and we have nuanced the discussion. We hope we have improved the quality of the manuscript by taking the reviewers’ suggestions into account.**

## **Abstract**

5. Thank you for reporting your abstract according to PRISMA for abstracts, following the PLOS Medicine abstract structure (Background, Methods and Findings, Conclusions)

We note reviewer #1 comments (below) and agree with the authors regarding combining the methods and findings section i-line with our formatting requirements.

- **Response:**

**We have left the `methods` and `findings´ sections combined in the abstract, in accordance with PLOS Medicine’s formatting recommendations.**

6. Line 41 – please define RR and 95% CI at first use.

- **Response:**

**We have revised the abstract accordingly.**

- **Changes in the manuscript:**
- **In the Abstract section (page 2):**

**“In Europe, migrant women had a higher risk of maternal mortality than native-born women (pooled *risk ratio [RR]*, 1.34; 95% *confidence interval [CI]*, 1.14, 1.58; p<0.001), but not in the USA or Australia.”**

7. Suggest the use of commas instead of hyphens (as these can be confused with reporting of negative values) to separate upper and lower bounds.

- **Response:**

**We have replaced hyphens by commas within confidence intervals throughout the manuscript.**

8. PLOS Medicine requests that where 95% CIs are reported p values are also reported. Please report a p <0.001 or where higher as p=0.002, for example. Suggest formatting statistical information as follows, “…(pooled RR 1.36; 95%CI [1.13-1.64]; p</=)…”.

- **Response:**

**We have added p values where 95% Cis are reported.**

- **Changes in the manuscript:**
- **In the Abstract section (page 2):**

**“In Europe, migrant women had a higher risk of maternal mortality than native-born women (pooled risk ratio [RR], 1.34; 95% confidence interval [CI], 1.14, 1.58; *p<0.001*), but not in the USA or Australia. Some subgroups of migrant women, including those born in sub-Saharan Africa (pooled RR, 2.91; 95%CI, 2.03, 4.15; *p<0.001*), Latin America and the Caribbean (pooled RR, 2.77; 95%CI, 1.43, 5.35; *p=0.002*) and Asia (pooled RR, 1.57, 95%CI, 1.09, 2.26; *p=0.01*) were at higher risk of maternal mortality than native-born women, but not those born in Europe or in the Middle East and North Africa.”**

9. Please include any important dependent variables that are adjusted for in the analyses.

- **Response:**

**We did not estimate adjusted risk ratios because our goal was to estimate the differential risk between migrant and native-born women. Our objective was therefore descriptive, as we think this is the first and essential step before considering causal mechanisms. We estimated an unadjusted risk ratio for each outcome with the number of events and of migrant and native-born women reported in each study.**

10. In the last sentence of the Abstract Methods and Findings section, please describe the main limitation(s) of the study's methodology.

- **Response:**

**We have added a sentence at the end of the Methods and Findings section of the abstract to acknowledge that our analysis did not take into account multiple social factors that may interact to determine maternal health. This limitation has also been added in the revised discussion section of the manuscript.**

- **Changes in the manuscript:**
- **In the Abstract section, end of the Methods and Findings subsection (page 2):**

***“We were unable to take into account other social factors that might interact with migrant status to determine maternal health because many of these data were unavailable.”***

11. Abstract Conclusions:

Please address the study implications without overreaching what can be concluded from the data; the phrase "In this study, we observed ..." may be useful.

Please interpret the study based on the results presented in the abstract, emphasizing what is new without overstating your conclusions.

Please avoid vague statements such as "these results have major implications for policy/clinical care". Mention only specific implications substantiated by the results.

When making your revision please ensure avoidance of any assertions of primacy.

- **Response:**

**We have revised the abstract Conclusions according to your recommendations.**

- **Changes in the manuscript:**
- **In the Abstract section, in the Conclusions subsection (pages 2 and 3):**

***“In this systematic review of the existing literature applying a single definition of "migrant” women, we found that* the differential risk of severe maternal outcomes in migrant versus native-born women in high income countries varied by host country and region of origin. *These results provide insights valuable for further exploring the causal mechanisms of these inequalities.”***

## **Author summary**

12. At this stage, we ask that you include a short, non-technical Author Summary of your research to make findings accessible to a wide audience that includes both scientists and non-scientists. The author summary should consist of 2-3 succinct bullet points under each of the following headings:

• Why Was This Study Done? Authors should reflect on what was known about the topic before the research was published and why the research was needed.

• What Did the Researchers Do and Find? Authors should briefly describe the study design that was used and the study’s major findings. Do include the headline numbers from the study, such as the sample size and key findings.

• What Do These Findings Mean? Authors should reflect on the new knowledge generated by the research and the implications for practice, research, policy, or public health. Authors should also consider how the interpretation of the study’s findings may be affected by the study limitations.

The Author Summary should immediately follow the Abstract in your revised manuscript. This text is subject to editorial change and should be distinct from the scientific abstract. Please see our author guidelines for more information: https://journals.plos.org/plosmedicine/s/revising-your-manuscript#loc-author-summary.

- **Response:**

**We have included the recommended Author Summary.**

- **Changes in the manuscript (pages 4 and 5): Author Summary section added:**
- **Why Was This Study Done?**

***“-	Some studies conducted in high-income countries report that the risk of maternal mortality and severe maternal morbidity is higher for migrant than native-born women, while other studies do not.***

***-	Whether this heterogeneity is related to differences in the definition and measurement of migration and of maternal outcomes or to real differences between settings remains unclear.”***

- **What Did the Researchers Do and Find?**

***“- In this literature review including 35 studies, we found that, in high income countries, the differential risk of severe maternal outcomes in migrant women, defined as born outside the host country, compared to native-born women, varied by the host country and the migrant women’s region of birth.***

***-	In Europe, migrant women were generally at higher risk of severe maternal outcomes than native-born women, whereas the risks for migrant women did not differ significantly from those for native-born women in United States of America or Australia.***

***-	Among migrant women, those born in sub-Saharan Africa, in Latin America and the Caribbean or in Asia were at higher risk of severe maternal outcomes than their native-born counterparts, while those born in Europe or in the Middle East and North Africa were not.”***

- **What Do These Findings Mean?**

***“-	When a single definition of “migrant” women was applied, the differential risk of severe maternal outcomes in migrant versus native-born women in high income countries varied by host country and region of origin.***

***-	This result provides insight valuable for further exploring these inequalities’ mechanisms.***

***-	Future studies should use harmonized definitions for all-cause and cause-specific severe maternal morbidity and take into account the multiple dimensions of the women’s social context.”***

## **Methods and results**

13. Please include details of how non-English language sources of studies were handled – see also methodological reviewer comments attached.

- **Response:**

**We searched Medline via PubMed, Embase and Cochrane Library without language restriction. All abstracts were available in English. After the first selection on abstracts, all relevant studies were in English.**

14. Could your study have searched databases more widely, would this have provided nay helpful additional information?

- **Response:**

**We searched Medline via PubMed and Embase without language restriction, following the Cochrane recommendations (Higgins JPT, Thomas J, Chandler J, Cumpston M, Li T, Page MJ, Welch VA (editors). Cochrane Handbook for Systematic Reviews of Interventions version 6.3 (updated February 2022). Cochrane, 2022. Available from www.training.cochrane.org/handbook). We considered that the most important databases regarding maternal health are published in medical journals, which are referenced in Medline and Embase.**

**We added the search of Cochrane Library for this update without language restriction with the same search algorithm as Medline via PubMed and Embase. It permits us to identify 16 records. Two reviewers independently screened first titles and abstracts. All of these were irrelevant and were excluded at the first selection.**

**We have added the literature search algorithm on Cochrane Library in a supplementary Table S3.**

**Other resources, such as Scopus, CINAHL, PsycInfo and ERIC do not include subjects on our topic.**

**And Google Scholar includes everything but lacks the ability to perform rigorous electronic searches, contrary to Medline and Embase.**

**We updated our search on April 18, 2023 and have changed the search dates in the manuscript, figures and supplementary material accordingly. This update yielded 3 additional studies that are now included in our manuscript:**

1. **Diguisto C, Saucedo M, Kallianidis A, Bloemenkamp K, Bødker B, Buoncristiano M, et al. Maternal mortality in eight European countries with enhanced surveillance systems: descriptive population based study. BMJ. 2022;e070621.**
2. **Jairam JA, Vigod SN, Siddiqi A, Guan J, Boblitz A, Wang X, et al. Severe Maternal Morbidity and Mortality Among Immigrant and Canadian-Born Women Residing Within Low-Income Neighborhoods in Ontario, Canada. JAMA Netw Open. 2023;6:e2256203.**
3. **Kallianidis AF, Schutte JM, Schuringa LEM, Beenakkers ICM, Bloemenkamp KWM, Braams-Lisman BAM, et al. Confidential enquiry into maternal deaths in the Netherlands, 2006-2018. Acta Obstet Gynecol Scand. 2022;101:441‑9.**

**Data from these 3 papers have been included in our meta-analysis when appropriate, and forest plots and pooled estimates have been updated accordingly.**

- **Changes in the manuscript:**
- **In the Abstract section, in the Methods and findings subsection (page 2):**

***“*Systematic literature review and meta-analysis using the MEDLINE/PubMed, EMBASE *and Cochrane Library* databases for the period from January 1, 1990 *to April 18, 2023.”***

- **In the Methods section, in the Search strategy and selection criteria subsection (page 8):**

***“*We searched Medline via PubMed, Embase *and Cochrane Library* without language restriction*.”***

- **In the Results section, in the Characteristics and methodological quality assessment subsection (page 11):**

***“*Of the *2290* records screened, *185* were relevant for full-text review and *39* were finally included (Fig 1 and *Table S4*).”**

- **Changes in the Figures:**
- **Figure 1. Study selection (page 1):**

Records identified from:

*Medline (n=1366)*

*Embase (n=2011)*

*Cochrane Library (n=16)*

Reference lists (n=30)

Records removed *before screening*:

Duplicate records removed *(n=1133)*

**Identification**

Records screened

*(N=2290)*

Records excluded

*(n=2105)*

**Screening**

Reports excluded *(n=146):*

Did not measure outcomes of interest *(n=60)*

Definition of *migrant women* not based on region of birth *(n=40)*

Non-original data (n=18)

Duplicate studies (n=14)

Host country women were not the comparison group (n=9)

Did not take place in a high-income country (n=4)

Did not provide sufficient information to calculate rates (n=1)

Reports assessed for eligibility

*(N=185)*

Reports included in review

*(N=39)*

**Included**

- **Changes in the Supplementary material:**
- **Table S3. Literature search algorithm on Cochrane Library (page 4):**

| ***Set#*** | ***Search term*** |
| --- | --- |
| *S1* | *(Migrant):ti,ab,kw OR (Migrants):ti,ab,kw OR (Immigrant):ti,ab,kw OR (Immigrants):ti,ab,kw OR (emigrant):ti,ab,kw OR (emigrants):ti,ab,kw OR (Foreign-born):ti,ab,kw OR (Foreign born):ti,ab,kw OR (Migration):ti,ab,kw OR (Migrations):ti,ab,kw OR (Immigration):ti,ab,kw OR (Immigrations):ti,ab,kw OR (Emigration and Immigration[mh]) OR (transients and migrants[mh]) OR (maternal country of birth):ti,ab,kw OR (maternal region of birth):ti,ab,kw OR (maternal place of birth):ti,ab,kw OR (legal status):ti,ab,kw OR (administrative status):ti,ab,kw OR (asylum seeker):ti,ab,kw OR (asylum seekers):ti,ab,kw OR (refugee):ti,ab,kw OR (refugees):ti,ab,kw OR (refugees[mh]) OR (expatriate):ti,ab,kw OR (expatriates):ti,ab,kw OR (exile):ti,ab,kw OR (exiles):ti,ab,kw* |
| *S2* | *(maternal mortality):ti,ab,kw OR ("maternal mortalities"):ti,ab,kw OR (maternal mortality[mh]) OR ("maternal death"):ti,ab,kw OR ("maternal deaths"):ti,ab,kw OR (maternal death[mh]) OR ("Pregnancy-related mortality"):ti,ab,kw OR ("Pregnancy-related mortalities"):ti,ab,kw OR ("Pregnancy-associated mortality"):ti,ab,kw OR ("Pregnancy-associated mortalities"):ti,ab,kw OR ("Maternal near-miss"):ti,ab,kw OR ("severe maternal morbidity"):ti,ab,kw OR ("severe maternal morbidities"):ti,ab,kw OR ("severe acute maternal morbidity"):ti,ab,kw OR ("severe acute maternal morbidities"):ti,ab,kw OR ("Obstetric hemorrhage"):ti,ab,kw OR ("Obstetric hemorrhages"):ti,ab,kw OR ("Obstetric haemorrhage"):ti,ab,kw OR ("Obstetric haemorrhages"):ti,ab,kw OR ("Postpartum hemorrhage"):ti,ab,kw OR ("Postpartum hemorrhages"):ti,ab,kw OR ("Postpartum haemorrhage"):ti,ab,kw OR ("Postpartum haemorrhages"):ti,ab,kw OR ("Postpartum hemorrhage"[mh]) OR (“peripartum hysterectomy”):ti,ab,kw OR (“peripartum hysterectomies”):ti,ab,kw OR (“peri partum hysterectomy”):ti,ab,kw OR (“peri partum hysterectomies”):ti,ab,kw OR (“pregnancy-related hysterectomy”):ti,ab,kw OR (Eclampsia):ti,ab,kw OR (Eclampsias):ti,ab,kw OR (Eclampsia[mh]) OR (Preeclampsia):ti,ab,kw OR (Preeclampsias):ti,ab,kw OR (“Pre eclampsia”):ti,ab,kw OR (“Pre eclampsias”):ti,ab,kw OR (“Pregnancy toxemia”):ti,ab,kw OR (“Pregnancy toxemias”):ti,ab,kw OR (“Toxemia of pregnancy”):ti,ab,kw OR (“Toxemia of pregnancies”):ti,ab,kw OR (Preeclampsia[mh]) OR ("Maternal sepsis"):ti,ab,kw OR (“uterine rupture”):ti,ab,kw OR (“uterine ruptures”):ti,ab,kw OR (uterine rupture[mh]) OR (“Maternal admission to the intensive care unit”):ti,ab,kw OR (“Maternal intensive care unit admission”):ti,ab,kw OR (“Maternal admission to an intensive care unit”):ti,ab,kw* |
| *S3* | *((maternal):ti,ab,kw AND (intensive care units[mh]))* |
| *S4* | *S1 AND (S2 OR S3)* |
| *S5* | *S4 AND (Observational study):ti,ab,kw* |

15. Line 167 – “…databases…” we agree with the methodological reviewer that seems rather an inappropriate description, suggest “…studies…”, as an alternative.

- **Response:**

**We have revised Table 1 and the manuscript according to your suggestion by changing “…databases…” for “…studies…”.**

- **Changes in the manuscript:**
- **In the Results section, in the Characteristics and methodological quality assessment subsection (page 11):**

**“Table 1 shows the characteristics of the 35 *studies* from Europe, Australia, the USA and Canada that furnished 39 *reports*. Because four *studies* yielded several *reports* covering different outcomes or different subgroups of migrant women, we included 39 *reports* overall. The median year of publication was 2017 (range 2008 to 2022). *Half the studies* used the ICD or procedure codes to define their outcomes (Tables 1 and S7). The assessment of the quality of the studies included is presented in the appendix (Table S6). According to the Newcastle-Ottawa scale score, only one study had a low methodological quality that limited the relevance of the subgroup analysis (Tables 1 and S6). Because only three *studies* contained information about legal status, we did not perform the corresponding subgroup analysis (Table 1).”**

- **In the Results section, in the Table 1 (pages 11 and 12):**

**“Table 1. Characteristics of included *studies***

| **Total = *35 studies*** | |  |
| --- | --- | --- |
| First year of inclusion period, median (min-max) | | 2007 (1988-2016) |
| Last year of inclusion period, median (min-max) | | 2012 (2005-2019) |
| **Host country, n (%)** | |  |
|  | Australia | 7 *(20)* |
|  | United States of America | 5 *(14)* |
|  | France | 4 *(11)* |
|  | Canada | *4 (11)* |
|  | Netherlands | *3 (9)* |
|  | United Kingdom | 3 (9) |
|  | Sweden | 2 (6) |
|  | Denmark | *2 (6)* |
|  | Germany | 2 (6) |
|  | *Norway* | *1 (3)* |
|  | Spain | 1 (3) |
|  | Italy | 1 (3) |
|  | Multi-country | 1 (3) |
| **Study population, n (%)** | |  |
|  | National | *21 (60)* |
|  | Regional | *9 (26)* |
|  | Mixed (National and Regional) | 1 (3) |
|  | Multicenter | 2 (6) |
|  | Single-center | 3 (9) |
| **Study design, n (%)** | |  |
|  | Cohort study | *19 (54)* |
|  | Cross-sectional study | *17 (49)* |
| **Source of information regarding *exposure of interest**, n (%)** | |  |
|  | *Birth and/or death certificates* | *29 (83)* |
|  | Birth register | 4 *(11)* |
|  | Interview | 1 (3) |
|  | Medical file | 1 (3) |
|  | Self-administered questionnaire | 1 (3) |
| **Legal status available, n (%)** | | 3 *(9)* |
| **Reported outcome, n (%)** | |  |
|  | Maternal mortality | *17 (49)* |
|  | All-cause severe maternal morbidity | *10 (29)* |
|  | Maternal near-miss | 3 (9) |
|  | Maternal intensive care unit admission | 5 *(14)* |
|  | Eclampsia | 4 *(11)* |
|  | Severe postpartum haemorrhage | 6 *(17)* |
|  | Severe sepsis | 4 *(11)* |
|  | Uterine rupture | 8 *(23)* |
| **Definition of outcome, n (%)** | |  |
|  | Clinical criteria specifically collected | *18 (51)* |
|  | Existing ICD or procedure codes | *18 (51)* |
| **Source of information regarding outcome, n (%)** | |  |
|  | Medico-administrative data | *15 (43)* |
|  | Medical file | *19 (54)* |
|  | Birth register | 2 (6) |
| **Newcastle-Ottawa scale score**, median (min-max) | | 7 (6-8) |

* *Migrant women* defined by region of birth”

16. As above, PLOS Medicine requests that where 95% CIs are reported p values are also reported. Please report a p <0.001 or where higher as p=0.002, for example. Please format statistical information as suggested above for the abstract.

Please replace the use of hyphens with commas to separate upper and lower bounds of 95% Cis

- **Response:**

**We have added p values where 95% CIs are reported.**

- **Changes in the manuscript:**
- **In the Results section, in the Maternal Mortality subsection (pages 12 and 13):**

**In European host countries, migrant women were at higher risk of maternal mortality than native-born women (pooled risk ratio [RR] 1.34; 95% confidence interval [CI], 1.14, 1.58; *p<0.001*; I²=58%; 95% CI; 18, 79), with risk ratios ranging from 0.77 (95% CI, 0.15, 3.80) to 2.01 (95% CI, 1.53, 2.65), whereas no excess risk was observed in the USA or Australia (p for interaction < 0.001) (Fig 2A). […] They included women born in sub-Saharan Africa (pooled RR, 2.91; 95% CI, 2.03, 4.15; *p<0.001*; I²=57%; 95% CI, 0, 83), in Latin America and the Caribbean (pooled RR, 2.77; 95% CI, 1.43, 5.35; *p=0.002*; I²=82%; 95% CI; 53, 93) and in Asia (pooled RR, 1.57; 95% CI, 1.09, 2.26; *p=0.01*; I²=31%; 95% CI, 0, 74).”**

- **In the Results section, in the All-cause severe maternal morbidity subsection (page 13):**

**“The pooled relative risk of all-cause severe maternal morbidity in migrant women compared to native-born women was 1.19 (95% CI, 0.95, 1.49; *p=0.1*; I²=87%; 95% CI, 69, 95) in European host countries and 0.99 (95% CI, 0.95, 1.03; *p=0.7*; I²=83%; 95% CI, 64, 92) in the USA and Australia (p for interaction < 0.001) (Fig 3A). Among migrant women, those born in sub-Saharan Africa (pooled RR, 1.54; 95% CI, 1.26, 1.88; *p<0.001*; I²=88%; 95% CI, 72, 95) or in Latin America and the Caribbean (pooled RR, 1.14; 95% CI, 1.05, 1.25; *p=0.003*; I²=51%; 95% CI, 0, 84) were at higher risk of all-cause severe maternal morbidity than their native-born counterparts, while those born in Europe or the Middle East and North Africa were not (p for interaction < 0.001).”**

## **Tables**

17. Table 1 – please clarify/revise the use of the term vital statistics.

- **Response:**

**The term vital statistics means the birth and/or death certificates. We have replaced the term “vital statistics” by “Birth and/or death certificates” in the Table 1.**

## **Figures**

18. Figure 1 – title: suggest “…study selection…”

- **Response:**

**We have changed the title of Figure 1 to “Study selection”**

- **Changes in the Figures:**
- **In Figure 1 (page 1):**

***“*Figure 1. *Study selection”***

19. As above, please ensure to revise figure titles to read “native-born”

- **Response:**

**We have changed this throughout the entire manuscript, including figures, tables and supplementary material.**

20. Throughout, please indicate in the figure captions whether your analyses are adjusted or unadjusted. Where adjusted analyses are presented, please also include unadjusted analyses for comparison and clearly state the factors that are adjusted for.

Please also clearly indicate the meaning of the dots and lines in the figures. All captions should clearly report the figure content without the reader needing to refer to the manuscript text.

- **Response:**

**We have edited all figure captions now indicating that our analyses are unadjusted and the meaning of the dots and lines in the figures.**

- **Changes in the Figures:**
- **In the Figures 2A, 2B, 3A and 3B (pages 2 to 5):**

***“Unadjusted risk ratios are random-effects estimates calculated by the DerSimonian and Laird method. The data markers show the unadjusted risk ratios (RR) with their 95% confidence intervals. The size of the data markers indicates the weight of the study. Diamonds show the pooled unadjusted risk ratios. The confidence interval is shown with lines.”***

- **In the Figures 4A, 4B, 4C, 4D, 4E and 4F (pages 6 to 11):**

***“The squares show the unadjusted risk ratios (RR) with their 95% confidence intervals. The size of the squares indicates the weight of the study. The confidence interval is shown with lines.”***

## **Discussion**

21. Please remove all sub-headings from the discussion such that the discussion reads as continuous prose.

- **Response:**

**We have removed all sub-headings from the discussion.**

22. Please present and organize the Discussion as follows: a short, clear summary of the article's findings; what the study adds to existing research and where and why the results may differ from previous research; strengths and limitations of the study; implications and next steps for research, clinical practice, and/or public policy; one-paragraph conclusion.

- **Response:**

**We have organized the Discussion as follows:**

1- **A short, clear summary of the article's findings**
2- **What the study adds to existing research and where and why the results may differ from previous research;**
3- **Strengths and limitations of the study;**
4- **Implications and next steps for research, clinical practice, and/or public policy;**
5- **One-paragraph conclusion.**

23. Line 230 – please avoid assertions of primacy which can be risky, suggest “…to our knowledge…”

- **Response:**

**We agree and have modified the sentence accordingly.**

- **Changes in the manuscript:**
- **In the Discussion section (line 328 page 17):**

**“*To our knowledge*, this is the first systematic review and meta-analysis focusing on the association of migrant women and native-born women in high income countries with various severe maternal outcomes and according to a single and consensual definition of migrant women.”**

## **References**

24. Please include an access date for web references.

- **Response:**

**We have included an access date for all web references (references 1, 2, 4, 23, 49 and 55).**

## **Supporting information**

25. Please ensure that figure/table captions clearly define all content for the reader without the need to refer to the text

- **Response:**

**We have revised all figure captions, indicating that our analyses are unadjusted and the meaning of the dots and lines in the figures.**

- **Changes in the Supplementary material:**
- **Footnotes of Figures S1 and S2 (pages 35 and 36):**

***“The squares show the unadjusted risk ratios (RR) with their 95% confidence intervals. The size of the squares indicates the weight of the study. The confidence interval is shown with lines.”***

- **Footnotes of Figures S3 and S4 (pages 37 and 38):**

***“Unadjusted risk ratios are random-effects estimates calculated by the DerSimonian and Laird method. The data markers show the unadjusted risk ratios (RR) with their 95% confidence intervals. The size of the data markers indicates the weight of the study. Diamonds show the pooled unadjusted risk ratios. The confidence interval is shown with lines.”***

- **Footnotes of Figures S5A and S5B (pages 39 and 40):**

***“SE = Standard Error; RR=Risk Ratio***

***The x-axis represents the unadjusted risk ratio between migrant and native-born women; the y-axis represents the standard error of the log risk ratio. Each plotted point represents a study. The vertical line represents the pooled risk ratio found in the meta-analysis.”***

26. PRISMA checklist – thank you for including the PRISMA checklist, please amend and refer to section and paragraph numbers rather than page (or line) numbers as these often in the event of publication.

- **Response:**

**We have edited the PRISMA checklist according to your guidelines by referring to section and paragraph numbers.**

27. Table S3 – in context of transparent data reporting, the editorial team hugely appreciated the inclusion of this table but it is not a requirement.

- **Response:**

**We would prefer to keep this Table as it provides useful information on source data to support our findings. We leave the editor the final choice as to whether this Table should be included in the main paper or in the Supplementary material.**

28. Table S5 – please clarify/revise the use of the term vital statistics

- **Response:**

**The term vital statistics means the birth and death certificates. We have replaced the term “vital statistics” by “Birth and/or death certificates” in Table S7.**

**Answers to Reviewers**

## **Reviewer #1**

1. The authors address an important public health issue. It is nicely written, apart from the numerous unnecessary Oxford-commas.

- **Response:**

**Thank you for your comment. We hope we have improved the quality of the manuscript by taking into account the editors’ and reviewers’ helpful suggestions. We have deleted all Oxford commas in the manuscript, except where the deletion could create ambiguity.**

2. I would be interested to hear from the authors whether ´inequities` are not better than `inequalities.

- **Response:**

**The term “inequalities” refers to the fact the different subgroups have different outcomes, in a descriptive approach; while the term “inequities” refer to the fact that individuals do not receive what they should according to their baseline needs, in a mechanistic approach.**

**We believe that our analysis describes inequalities rather than inequities, since our objective was not to document access to or quality of care.**

3. I would separate `methods` from `findings´ in the abstract.

- **Response:**

**As the editor pointed out, we did not separated `methods` from `findings´ in the abstract, because we followed PLOS Medicine’s formatting guidelines.**

4. My main problem is with the definition of migrants as those who were not born in the host country. What then do we do with `women`who are born in the host countries`but with parents from the country of origin? Are they not migrants anymore?

That is different what we did with similar studies in the Netherlands. The definition also confused me in line 45, 282, 296 and 312 and that should be addressed in a revised version.

- **Response:**

**In this systematic review, we used the definition from the International Organization for Migration (IOM) (McAuliffe, M., & Triandafyllidou, A. (2021). World Migration Report 2022 2022. International Organization for Migration (IOM), Geneva. Available from:** [**https://www.iom.int/**](https://www.iom.int/)**) and recommended by the Reproductive Outcomes and Migration project, in collaboration with the EURO-PERISTAT project, to study migrants’ perinatal health. In this definition, women who are born in the host countries from parents born in another country are not considered migrant women.**

**This definition of migrants strictly limited to those who were not born in the host country offers the advantage of targeting a homogeneous group of first-generation migrants. However, it does not include women who would be defined as second-generation migrants. This specific group is interesting to study, but databases with available data on the parents’ and grandparents’ countries of birth are still too rare for a synthesis of their results to be useful. We now have discussed this point in the revised version.**

- **Changes in the manuscript:**
- **In the Discussion section (Line 340-347 pages 17 and 18):**

***“This definition of migrant women, as being born outside the host country, is the one used by the International Organization for Migration [70] and recommended by the Reproductive Outcomes and Migration collaboration and the EURO-PERISTAT project, to study migrants’ perinatal health [36]. It offers the advantage of targeting a homogeneous group of first-generation migrants. It does not, however, include women who would be defined as second-generation migrants. This specific group is interesting to study, but databases with available data on the parents’ and grandparents’ countries of birth are still too rare for a synthesis of their results to be useful.”***

5. I agree with you that you cannot consider all migrants (called global migrants) as coming from the same background and consider them as coming from different regions is a helpful concept.

- **Response:**

**Thank you for your comment.**

6. In abstract, line 50/51 it is unclear when you state `there is a need .... and to customize related health policies in each context.` Customized to what?

- **Response:**

**At editors’ request, we have revised the abstract’s conclusions to avoid overreaching and vague statements concerning study implications.**

- **Changes in the manuscript:**
- **In the Abstract section, in the Conclusions subsection (pages 2 and 3):**

***“In this systematic review of the existing literature applying a single definition of "migrant” women,* we found that the differential risk of severe maternal outcomes in migrant versus native-born women in high income countries varied by host country and region of origin. *These results provide insights valuable for further exploring the causal mechanisms of these inequalities.”***

7. In line 51/52 you excluded maternal mental health problems from the review, but did not give an argument for that. It is, however, a frequent outcome of childbirth, and needs a reason for exclusion.

- **Response:**

**We agree with your comment that maternal mental health issues are a frequent outcome of childbirth, particularly for migrant women. However, we believe that this topic requires a specific systematic review, especially as the definition of mental health is very specific and complex. In addition, we noticed that a systematic review on this topic was already registered by Kerrie Stevenson (PROSPERO, CRD42021226291). This work was indeed published in the Lancet Public Health in March 2023 (Stevenson K, Fellmeth G, Edwards S, Calvert C, Bennett P, Campbell OMR, et al. The global burden of perinatal common mental health disorders and substance use among migrant women: a systematic review and meta-analysis. Lancet Public Health. 2023;8:e203‑16). We have mentioned this reason for exclusion in the manuscript.**

- **Changes in the manuscript:**
- **In the Methods section, in the Search strategy and selection criteria subsection (page 9):**

***“This review did not consider maternal mental health outcomes because we believe they require a specific assessment and because a systematic review on this topic was registered as underway when we started (PROSPERO, CRD42021226291) and finally published [48].”***

8. I did find in the figures one of our dutch papers being referenced as `Zwart and Van Hanegem`±I saw Zwart´s BJOG paper in the reference list but not the paper with Van Hanegem as the first autor (Acta Obstet Gynecol Scand)?

- **Response:**

**We have edited the references listed in forest plots by mentioning separately Zwart et al. and Van Hanegem et al.**

**We have also added a supplementary material listing all the included studies (Table S4).**

## **Reviewer #2**

1. Page 5 If any of the articles were in languages other than English then somewhere we need to know how they were translated or otherwise dealt with.

- **Response:**

**We searched Medline via PubMed and Embase without language restriction. All abstracts were available in English. After the first selection on abstract, all relevant studies were in English. We therefore did not have to translate articles in languages other than English.**

2. Page 6 If authors were asked to supply extra data then I think that (a) we should be told which items we are not going to be able to find in the original publications (b) the people who supplied it should be acknowledged. If this is what the list on page 15 is then perhaps state there why they are being acknowledged. That would address point (b) but not (a).

- **Response:**

**Exactly, the list in Acknowledgements section represents all authors who supplied extra data. We have added the specific assistance for which they are being acknowledged. We have also edited the Methods section, data analysis subsection.**

- **Changes in the manuscript:**
- **In the Methods section, in the Data analysis subsection (page 9):**

**“We contacted corresponding authors *to obtain additional data not available in the original publication.”***

- **In the Acknowledgements section (pages 20 and 21):**

**“The authors would like to thank Annika Esscher and Birgitta Essén (both from the Department of Women's and Children's Health, Uppsala University, Uppsala, Sweden) *who supplied data about the number of deaths and maternal mortality ratios for women of reproductive age born in low-, middle- and high-income countries and in Sweden*; Ayesha Siddiqui (Université de Paris, CRESS, Obstetrical Perinatal and Pediatric Epidemiology Research Team, EPOPé, INSERM, INRA, Paris, France) *who supplied data about the total number of women diagnosed with severe preeclampsia by distinguishing the women born in France from those born elsewhere in Europe*; Baiju Shah (Sunnybrook Health Sciences Centre, Toronto, ON, Canada; Sunnybrook Research Institute, Toronto, ON, Canada; Department of Medicine, University of Toronto, ON, Canada) *who supplied data for the total number of women with and without preeclampsia/eclampsia among each of the nonimmigrant, refugee and other-immigrant groups*; Birgit Reime (Faculty of Health Safety Society, Furtwangen University, Furtwangen, Germany) *who supplied data for the total number of women diagnosed with cause-specific severe maternal morbidity among native-born and migrant women in Germany between 2001 and 2007*; Jessica Turner (Mater Research Institute, University of Queensland, Brisbane, Australia) *who supplied data for the total number of women diagnosed with massive postpartum hemorrhage and ICU admission among refugees and women born in Australia*; Joost Zwart (Department of Obstetrics and Gynaecology, Deventer Ziekenhuis, 7416 SE Deventer, The Netherlands) and Jos van Roosmalen (Department of Obstetrics and Gynaecology, Leiden University Medical Center, 2300 RC Leiden, The Netherlands) *who supplied data for the total number of deliveries among Western and non-Western migrant women*; Kjersti Sletten Bakken (Centre for Intervention Science in Maternal and Child Health [CISMAC], Department of Global Public Health and Primary Care, University of Bergen) *who supplied data about the total number of women diagnosed with preeclampsia/eclampsia and HELLP syndrome among first-generation immigrants, second-generation immigrants and native-Norwegian-born women*; Marcelo Urquia (Department of Community Health Sciences, University of Manitoba Faculty of Health Sciences, Winnipeg, Manitoba, Canada) *who supplied data about the total number of women diagnosed with eclampsia according to their region of birth*; Miguel-Angel Luque-Fernandez (Department of Non-Communicable Disease Epidemiology, Cancer Survival Group, London School of Hygiene and Tropical Medicine, London; and Department of Non-Communicable Disease and Cancer Epidemiology, Instituto de Investigacion Biosanitaria de Granada (Ibs.GRANADA), University of Granada, Granada, Spain) *who supplied data about the total number of live births in Spain and in Europe excluding Spain*; Stephanie Leonard (Department of Obstetrics and Gynecology, Stanford University, Palo Alto, CA; California Maternal Quality Care Collaborative, Stanford University, Palo Alto, CA) *who supplied data on the total number of women with severe maternal morbidity, and with non-transfusion severe maternal morbidity in native-born women and migrant women. We would also like to thank Jo Ann Cahn for her English editorial assistance*.”**

3. Page 6 How did the authors score studies in which they had been involved?

I assume that the Eslier who is an author on some of the references is the same as the current first author. Since quality scores do not play a major part subsequently it would be acceptable not to score those studies. Finding someone else to do it who does not have a conflict of interest would be almost impossible.

- **Response:**

**We agree with your comment. We have tried to limit this conflict of interest by having one of the two reviewers not involved in the studies cited. This conflict of interest is clearly identifiable as we have listed all references included in order to be as transparent as possible with the readers.**

4. Page 7 Confidence intervals for I2 would also be helpful to reveal our uncertainty about the heterogeneity. I know we did not use to use them but I am persuaded by Ioannidis et al. (2007) that we should. For instance in Figure 2A for the first analysis I2 = 68 (32 to 85) which is quite imprecise although the second one is more precise 93 (88 to 96).

- **Response:**

**Thank you for this reference that supports the need to report I² with 95% confidence intervals, a point discussed in the literature, particularly in cases of limited number of events (Thorlund et al. 2012. PLOS One). We have calculated and added confidence intervals for all I² values in the revised manuscript and edited the Methods section accordingly.**

**Ref: Higgins JP, Thompson S. Quantifying heterogeneity in a meta-analysis. Statistics in Medicine. 2002;21:1539–1558.**

- **Changes in the manuscript:**
- **In the Methods section, in the Data analysis subsection (page 10):**

**“Between-study heterogeneity was explored by visual examination of the forest plots and by the heterogeneity test and *I² statistic with its 95% confidence interval [51,52].”***

5. Page 7 I do not quite understand the point about heterogeneity here. Do the authors mean that they decided on scientific grounds that there would be heterogeneity and so decided to use random effect models (which seems to me the way to go) or did they do it dependent on observed statistical heterogeneity? It looks like the former but it was not clear to me.

- **Response:**

**We confirm that we decided to use random effect models because of expected heterogeneity on scientific grounds. We have edited the Methods section to clarify this point.**

- **Changes in the manuscript:**
- **In the Methods section, in the Data analysis subsection (page 10):**

**“*Because heterogeneity was expected on scientific grounds,* we used random-effects meta-analysis models with the DerSimonian and Laird method [51].”**

6. Page 8 Providing a list of excluded studies is helpful but seldom done.

- **Response:**

**Thank you. In context of transparent data reporting, we decided to provide this list of excluded studies.**

7. Page 8 and elsewhere I wonder whether databases is the best word here? It surprised me on first reading. Would studies or datasets be acceptable alternatives?

- **Response:**

**We have revised Table 1 and the manuscript according to your suggestion by replacing “…databases…” by “…studies…”.**

- **Changes in the manuscript:**
- **In the Results section, in the Characteristics and methodological quality assessment subsection (page 11):**

**“Table 1 shows the characteristics of the 35 *studies* from Europe, Australia, the USA and Canada that furnished 39 *reports*. Because four *studies* yielded several *reports* covering different outcomes or different subgroups of migrant women, we included 39 *reports* overall. The median year of publication was 2017 (range 2008 to 2022). *Half the studies* used the ICD or procedure codes to define their outcomes (Tables 1 and S7). The assessment of the quality of the studies included is presented in the appendix (Table S6). According to the Newcastle-Ottawa scale score, only one study had a low methodological quality that limited the relevance of the subgroup analysis (Tables 1 and S6). Because only three studies contained information about legal status, we did not perform the corresponding subgroup analysis (Table 1).”**

- **In the Results section, in the Table 1 (pages 11 and 12):**

**“Table 1. Characteristics of included *studies***

| **Total = *35 studies*** | |  |
| --- | --- | --- |
| First year of inclusion period, median (min-max) | | 2007 (1988-2016) |
| Last year of inclusion period, median (min-max) | | 2012 (2005-2019) |
| **Host country, n (%)** | |  |
|  | Australia | 7 *(20)* |
|  | United States of America | 5 *(14)* |
|  | France | 4 *(11)* |
|  | Canada | *4 (11)* |
|  | Netherlands | *3 (9)* |
|  | United Kingdom | 3 (9) |
|  | Sweden | 2 (6) |
|  | Denmark | *2 (6)* |
|  | Germany | 2 (6) |
|  | *Norway* | *1 (3)* |
|  | Spain | 1 (3) |
|  | Italy | 1 (3) |
|  | Multi-country | 1 (3) |
| **Study population, n (%)** | |  |
|  | National | *21 (60)* |
|  | Regional | *9 (26)* |
|  | Mixed (National and Regional) | 1 (3) |
|  | Multicenter | 2 (6) |
|  | Single-center | 3 (9) |
| **Study design, n (%)** | |  |
|  | Cohort study | *19 (54)* |
|  | Cross-sectional study | *17 (49)* |
| **Source of information regarding *exposure of interest**, n (%)** | |  |
|  | *Birth and/or death certificates* | *29 (83)* |
|  | Birth register | 4 *(11)* |
|  | Interview | 1 (3) |
|  | Medical file | 1 (3) |
|  | Self-administered questionnaire | 1 (3) |
| **Legal status available, n (%)** | | 3 *(9)* |
| **Reported outcome, n (%)** | |  |
|  | Maternal mortality | *17 (49)* |
|  | All-cause severe maternal morbidity | *10 (29)* |
|  | Maternal near-miss | 3 (9) |
|  | Maternal intensive care unit admission | 5 *(14)* |
|  | Eclampsia | 4 *(11)* |
|  | Severe postpartum haemorrhage | 6 *(17)* |
|  | Severe sepsis | 4 *(11)* |
|  | Uterine rupture | 8 *(23)* |
| **Definition of outcome, n (%)** | |  |
|  | Clinical criteria specifically collected | *18 (51)* |
|  | Existing ICD or procedure codes | *18 (51)* |
| **Source of information regarding outcome, n (%)** | |  |
|  | Medico-administrative data | *15 (43)* |
|  | Medical file | *19 (54)* |
|  | Birth register | 2 (6) |
| **Newcastle-Ottawa scale score**, median (min-max) | | 7 (6-8) |

* *Migrant women* defined by region of birth”

8. Page 9 What does vital statistics mean here? For me it means the central records kept of births and deaths and the publications which national statistics agencies produce from them but that does not seem relevant here.

- **Response:**

**The term vital statistics means the birth and/or death certificates. We have replaced the term “vital statistics” by “Birth and/or death certificates” in the Table 1.**

9. Page 11 I think what Figures 5a and 5b show is the limited value of funnel plots when there is high heterogeneity (as the authors themselves suggest). The plots do not present any concerns.

- **Response:**

**Thank you for your comment.**

10. Table S5 Some of the entries in the last column are not migrants place of birth as stated in the heading. Specifically Creaga has ethnicity not place of birth, Esscher has income classification not region as does Wahlberg.

Table S5 Are the studies which have not specified as place of birth really capable of being included in this review?

- **Response:**

**Thank you for this comment. We confirm that all studies, including those by Creanga and Esscher, met all inclusion criteria, and in particular the definition of migration as having been born outside the country.**

**Some studies also had additional information on ethnicity, which we decided to mention in our initial manuscript. However, we agree with the reviewer that this was confusing and therefore decided to remove the last column’s content when it did not concern the migrant women’s place of birth.**

11. Table S5 In the first two Knight studies the most numerous group was women born in Poland (which did not surprise me) but in the third study they do not seem to appear at all. Is this really the case?

- **Response:**

**The last column of Table S7 shows the migrant women's place of birth and its proportion regarding all migrant women. We contacted the authors and they responded was that they are not allowed to publish figures for countries of birth with 2 or fewer deaths, which is why Poland does not appear in the 2020 report. So the total number of births concerning women born in Poland is not available. Although migrant women born in Poland represent a large proportion of migrant women, the number of deaths among these women is low compared to other subgroups of migrant women (3 deaths between 2013 and 2015, and no more than 2 between 2016 and 2018).**

12. Multi–level nature of studies

The individual studies represented in the data consist of several sets which clearly come from the same group, using similar methods, and studying similar groups of women. Since these are not independent estimates one of the assumptions underlying meta–analysis, independence of the primary studies, seems hard to justify. There are various approaches to dealing with this like multi–level meta–analysis, aggregation of studies from the same source. At a minimum the problem needs noting and discussing.

- **Response:**

**We agree with your comment concerning the problem of independence of the primary studies in case of cumulating data on individual studies with similar groups of women. However, we have aggregated together all the studies reporting data from the same database. This was the case for Zwart et al. 2008; Zwart et al. 2010; Zwart et al. 2011; Van Hanegem et al. 2011 that came from the same database. We considered these reports as a single study in meta-analyses. We did not do so with the repeated reports from the UK, Australia and France because it corresponded to different samples and different periods. Based on your comment, we conducted a post hoc sensitivity analysis combining data from these repeated reports to get a single estimation from the UK, from Australia and from France for maternal mortality. This had no impact on the results with very similar results. We wonder whether we should include this sensitivity analysis on the manuscript because it was not planned and because it showed very similar results but if you think we should do so, we would happy to present these additional results.**

**Figure 1. Maternal mortality in migrant women and native-born women - post hoc sensitivity analysis combining data from repeated reports**

CI=confidence interval; IV=inverse variance; df=degrees of freedom

The squares show the unadjusted risk ratios (RR) with their 95 % confidence intervals. The size of the squares indicates the weight of the study. The confidence interval is shown with lines.

**Figure 2. Maternal mortality in migrant women and native-born women, stratified by host country - post hoc sensitivity analysis combining data from repeated reports**

CI=confidence interval; IV=inverse variance; df=degrees of freedom

Unadjusted Risk ratios are random-effects estimates calculated by the DerSimonian and Laird method. The data markers show the unadjusted risk ratios (RR) with their 95 % confidence intervals. The size of the data markers indicates the weight of the study. Diamonds show the pooled unadjusted risk ratios. The confidence interval is shown with lines.

**Figure 3. Maternal mortality in migrant women and native-born women, stratified by migrant women’s region of birth - post hoc sensitivity analysis combining data from repeated reports**

CI=confidence interval; IV=inverse variance; df=degrees of freedom

Unadjusted Risk ratios are random-effects estimates calculated by the DerSimonian and Laird method. The data markers show the unadjusted risk ratios (RR) with their 95 % confidence intervals. The size of the data markers indicates the weight of the study. Diamonds show the pooled unadjusted risk ratios. The confidence interval is shown with lines.

13. Host country and region are not independent

Differences found between host countries are confounded by differences between the regions from which the women migrated. I would suggest that we need a meta–regression of all the studies with host country and birth region as moderators to attempt to separate the effects. This would also give us an estimate of the differences between levels which presenting by subset does not do.

- **Response:**

**We acknowledge that host country and region are not independent but we think that a meta-regression of all the studies with host country and birth region as moderators, although theoretically interesting, would not be a good option here. Meta-regression is a weighted linear regression with the studies as the unit of analysis. However, the host country and the migrant women’s region of birth are closely related in the set of studies we found. There will thus be a problem of collinearity in the model, which is likely to bias the estimates.**

## **Reviewer #3**

1. Why did authors not used a method o describe the overall certainty of evidence such as GRADE methodology. Authors seems to focus on results of meta-analysis which is one piece of information and it needs to be considered with aspects of evidence such as type of study, risk of bias, heterogeneity of the evidence, indirectness and precision of summary estimates.

- **Response:**

**The GRADE methodology is a systematic approach for making clinical practice recommendation (Guyatt et al. GRADE: an emerging consensus on rating quality of evidence and strength of recommendations. BMJ 2008), and thus does not appear to us to be appropriate here.**

**We used the Newcastle–Ottawa scale for observational studies to assess the methodological quality of each study for the following items: selection of study groups, comparability of groups and ascertainment of the outcome of interest.**

2. Why did authors not report an overall risk of maternal mortality in high income country irrespective of region in high income country?

- **Response:**

**Our approach shows that the important heterogeneity between host countries means that a global risk estimate is necessarily misleading. Our findings demonstrate that reporting this overall risk of maternal mortality, regardless of region, in high income countries is neither advisable nor instructive.**

3. Authors report that risk of maternal mortality is high in immigrant mothers in Europe based on RR 1.36 (1.13-1.64). This mean a relative increase risk of about 36 %. What is the absolute risk per 100, 000 or number need to harm? This is important as the absolute risk might be small as overall the maternal mortality is not a common event.

- **Response:**

**We agree that maternal mortality is a rare event with a very low absolute risk, as appears clearly through the numbers of women in numerators and denominators shown in the forest plots.**

**However, we think that explicitly calculating these absolute risks in the perspective of describing inequalities is not useful, given the wide variations between countries in maternal mortality rates among native-born women.**

**Finally, the use of the number needed to harm does not appear relevant in the context of non-interventional studies.**

## **Reviewer #4**

1. However, I do believe that the research question and analysis are limited by the unidimensional comparison of foreign-born vs native-born women globally. Maternal mortality/morbidity is determined by complex interactions of a wide range of (social) determinants which need to be integrated in (intersectional) analyses to advance the current state of knowledge on this topic. Especially with regards to the Americas, comparing "native" to "foreign"born populations does not do justice to the colonial history, migration background of now 'native' populations etc. In line with this critique on the comparisons made, I believe that exclusion of studies before 1990 only has a very small impact on limiting heterogeneity in migrants' characteristics and integration policies.

Therefore, at least in the discussion of the findings, more attention needs to be given to the fact that comparing the umbrella term of migrants to the umbrella term of natives - without considering race/ethnicity of native groups as well, along with other determinants and their interactions - has only limited value to draw conclusions regarding the causal relationships between migration, ethnicity or any other variable and outcomes. For example, the authors could rephrase claims such as "the risk of severe maternal complications for mothers in the USA/Australia appears to be related more to race/ethnicity than to migration status".

- **Response:**

**We agree with the view that using migration as the only variable for characterizing exposure is insufficient for understanding the causal mechanisms of social inequalities in health. Maternal health is in fact determined by the complex interaction of multiple social factors that should ideally be integrated into the analyses. This systematic review, which aims to summarize the inequalities in maternal health between migrant and native-born women by introducing the women's place of birth and the host country, advances our understanding of the mechanisms. The introduction of other factors such as race/ethnicity, migrant women’s administrative status and economic factors is necessary to advance our understanding of the mechanisms at stake and should be recommended for future analyses of the association between migration and maternal health. We have added 2 sections in the revised discussion to expand the critical reflections on these aspects.**

- **Changes in the manuscript:**
- **In the Discussion section (lines 283-286 page 15):**

***“Thus, in these contexts, the comparison between "native" and "foreign-born" populations does not take into account the colonial history and migratory backgrounds of the now "native" populations that played a role in determining these groups’ social position and their health status.”***

- **In the Discussion section (lines 316-327 pages 16-17):**

***“An approach that focuses on the migration issue and takes the fact of being born abroad as the only exposure variable cannot by itself provide an insight into the mechanisms of social inequalities in health between migrant and native-born women. This approach is necessary and in our opinion justifies the studies thus far conducted in this way and included in this systematic review. It cannot, however, be sufficient. Maternal health is influenced by the complex interaction of multiple social factors. Among these factors, socioeconomic determinants, such as migrant women’s administrative status, should be considered and analyzed as potential intermediate factors. Race/ethnicity, a major determinant in contexts such as the USA or Australia, even when migration is the main focus of the studies, should also be collected and included in the analysis to enable multidimensional descriptions of the groups being compared and provide deeper insight to the causal relationships with health outcomes.”***

2. The authors should also be conscious of consistent use of all terms throughout the manuscript (for example, earlier in the paper they refer to "migration status" meaning "legal status", but in this specific statement migration status seems to refer to the fact that someone is a first-generation migrant).

- **Response:**

**We agree with your comment. We have edited all terms throughout the manuscript for consistency.**

3. With expanded critical reflections in the discussion of the findings, I believe the article to be of interest to clinicians and policymakers in the field of perinatal healthcare, as well as to researchers in the field of migration-related health inequities.

- **Response:**

**Thank you for your positive comment.** **We have expanded critical reflections in the revised discussion in answer to comment 1.**

4. Other points of concern for minor revisions:

- In the presentation of the finding concerning differential risk of severe outcomes in women from different regions of origin, it is now unclear that this analysis was performed for studies both in USA and Europe. From the reading of the results, the reader could assume that this difference only concerns studies from Europe (given that no differences were found between migrants-natives in USA without taking country of birth into account).

- **Response:**

**Subgroup analyses according to migrant women’s region of birth were performed including all available data from high income countries. However, the great majority of studies providing the information on the migrant women‘s country of birth were from European countries. This is why the forest plots by country of birth appear to include only European countries.**

5. The manuscript is generally well-organized and written clearly enough to be accessible, although it would benefit from a thorough language check by a native English speaker given a number of (relatively minor) mistakes that cumulatively do compromise easy reading of the review.

- **Response:**

**Thank you for your comment. The initial manuscript was edited by a professional native English-speaking biomedical editor and translator, who has reviewed this version before resubmission.**
