# Supplementary material for: Association between migration and severe maternal outcomes in high-income countries: Systematic review and meta-analysis
Source: PLoS Med. 2023 Jun 22;20(6):e1004257. doi: 10.1371/journal.pmed.1004257 (PMC10328365; doi:10.1371/journal.pmed.1004257)
Supplement: Attachment — Submitted filename: Eslier_Answers to reviewers_R3.docx [file pmed.1004257.s016.docx]

Manuscript number: PMEDICINE-D-22-03069R2

Title: Association between migration and severe maternal outcomes in high income countries: systematic review and meta-analysis

Responses to reviewers

**Answers to the Editors**

## **General**

1. The finding of no difference in outcomes in the US (or Australia) is interesting and surprising. How different is the benchmark across these countries i.e. are maternal outcomes for native-born women worse in the US (and/or Australia) compared to outcomes for native-born women in Europe, for example? Please consider how the results are framed/compared in this context.

- **Response:**

**We agree that the finding of no difference in outcomes between migrant women and natives in the USA, Canada or Australia is interesting. However, this finding needs to be put in perspective with the baseline maternal mortality or severe morbidity in native women in these regions compared to European countries. Indeed, the maternal mortality ratio is more than twice as high in native-born women in the USA than in native-born women in Europe (for example, 20.8 in the US versus 8.9 in France per 100,000 live births); this is also true for severe maternal morbidity with the limitation of definition heterogeneity across countries.**

**In our manuscript, the mortality and morbidity rates in native women can be calculated in the different host countries from the numbers indicated in the forest plots for readers who would like to compare them. This point was already raised in the discussion (page 15, lines 276-282) as follows:**

**“In the USA and Australia, the groups with the highest maternal mortality and morbidity rates are, respectively, Black [53] and indigenous [54–57] women, both included in the native-born reference group. The maternal mortality ratio is from three to six times higher in Black than in White women in the USA [58] and in indigenous versus non-indigenous women in Australia [54–57]. Furthermore, the overall maternal mortality ratio in the USA is more than twice as high as in Europe and this ratio for native-born US White women is double that for native-born Europeans [58–60].”**

## **Abstract and author summary**

2. It would be helpful to indicate that the studies included were conducted at a national level.

- **Response:**

**Actually, as described in Table 1 and detailed in Table S6, a majority of studies (60%) are national, others are regional (26%) and a minority are centre-based. This information is thus available for readers.**

## **Author summary**

3. Line 84 – ‘This result provides insight valuable for further exploring these inequalities’ mechanisms.’ Suggest instead, ‘These data highlight the need to further explore the mechanisms underlying these inequities.’ Or similar.

- **Response:**

**We have revised the abstract Conclusions and Author summary according to your suggestion.**

- **Changes in the manuscript:**
- **In the Abstract section, in the Conclusions subsection (page 3):**

**“*These data highlight the need to further explore the mechanisms underlying these inequities*.”**

- **In the Author summary section, in the “What Do These Findings Mean?” subsection (page 4):**

**“-*These data highlight the need to further explore the mechanisms underlying these inequities.”***

4. Line 86 – the latter part of this statement is rather vague ‘…take into account the multiple dimensions of the women’s social context’ please revise.

- **Response:**

**We have edited the Author summary section to clarify this point.**

- **Changes in the manuscript:**
- **In the Author summary section, in the “What Do These Findings Mean?” subsection (page 5):**

**“- Future studies should use harmonized definitions for all-cause and cause-specific severe maternal morbidity and take into account *other social factors such as race/ethnicity, migrant women’s administrative status and economic factors that may interact with migration to understand the inequalities in maternal health between migrant and native-born women.”***

## **Tables**

5. Table S3 – please feel free to include this table with the manuscript as supporting information.

- **Response:**

**Thank you for your suggestion. We have kept this Table as Table S5.**

## **References**

6. Ref 38 line 523 – what does the asterisk represent here? Can it be removed?

- **Response:**

**This asterisk is a mistake. We have removed it.**

7. Ref 67 line 608 – what to the 3 stars represent here? Can they be removed?

- **Response:**

**These 3 stars are citation errors with Embase. We have removed them.**

## **Social media**

21. If not already done so, to help us extend the reach of your research, please detail any Twitter handles you wish to be included when we tweet this paper (including your own, your co-authors’, your institution, funder, or lab) in the manuscript submission form when you re-submit the manuscript.

- **Response:**

**Thank you for your help to extend the reach of our study. We have added @Inserm, @Epope_Inserm, @univ_paris_cite, @GHPSJ as Twitter handles we wish to be included when we have submitted the manuscript.**

**Answers to Reviewers**

## **Reviewer #1**

1. The authors have undertaken a huge job to review all the literature in relation to migration and maternal health and bring together these data in a nice review. There is only one issue I still have, because my earlier question was not answered. I have very well understood their defintion of migrant and native women. The Dutch data, however, are still a bit different, because in our papers a woman born in the Netherlands but with one or two parents born in Marocco is still considered as a migrant woman. Only in the paper about asylumseekers the migrants cannot be born in the Netherlands, so they all confirm with the definition used in the paper. I cannot assess this issue for the other studies. But I would suggest to include this as a limitation, at least for our dutch studies. Jos van Roosmalen.

- **Response:**

**We agree with this comment and confirm that only the Dutch data have a bit different definition of migrant women. We still have included these data because the definition is based on the country of birth, either of the woman herself or of her parents. We have now mentioned this specificity as a footnote in table S6 and in the revised discussion section.**

- **Changes in the manuscript:**
- **In the Discussion section (pages 17-18, lines 345-351):**

**“*We were able to apply this definition for all included studies except for some Dutch studies where we cannot exclude that some second-generation migrant women were included in the migrant women group. This definition offers the advantage of targeting a homogeneous group of first-generation migrants. Second-generation migrant women constitute* a specific group interesting to study, but databases with available data on the parents’ and grandparents’ countries of birth are still too rare for a synthesis of their results to be useful.”**

- **In the Table S6 (footnote):**

**“**Some second-generation migrant women might have been included in the migrant women group.”***

## **Reviewer #2**

1. The authors have addressed all my points but there is one minor issue remaining.

I suggested performing a meta-regression using both host country and birth region as moderators. I did not find the response very convincing. They will no doubt be related, that is the point, but I do not see that the analysis is necessarily fatally flawed a priori. I would suggest trying it and seeing what happens or at least expand the limitations section to indicate that the relationship with host country and the relationship with birth region may be saying the same thing twice.

Michael Dewey.

- **Response:**

**We acknowledge that better studying the separate effect of host country and maternal birth region is of interest but we believe that a meta-regression with host country and birth region as moderators would raise several issues. First, as we previously indicated, the host country and the migrant women’s region of birth are closely related in the set of studies we found, resulting in collinearity in the model, likely to bias the estimates.**

**In addition, concerning maternal mortality, data on maternal region of birth was only available for studies performed in Europe. No study carried out in the USA or Australia reported data on maternal region of birth. Therefore, this meta-regression could not be made for maternal mortality.**

**A last issue is the fact that meta-regression should be conducted on study-level covariates (Thompson SG, Higgins JPT. How should meta-regression analyses be undertaken and interpreted? Stat Med. 2002;21:1559‑73). This is the case for the host country but not for maternal region of birth, which is a patient-level characteristic. Actually, in the included studies, there were different maternal regions of birth summarized with percentages. As highlighted by Thompson and Higgins, “the relationship with patient averages across trials may not be the same as the relationship for patients within trials”, which makes the interpretation of such analyses potentially misleading in case of patient-level covariates.**

**We have mentioned this limitation in the revised discussion section.**

- **Changes in the manuscript:**
- **In the Discussion section (page 18, lines 352-357):**

**“*In order to explore between-study heterogeneity, we decided to perform subgroup analyses instead of meta-regression with host country and region of birth as moderators because these characteristics are closely related in the set of studies we found, resulting in collinearity in the model, likely to bias the estimates. As a consequence, we cannot totally rule out that host country and region of birth actually characterized the same subgroups of migrant women*.”**

## **Reviewer #4**

1. Dear authors,

It was a pleasure to review your revised manuscript. I believe you improved the manuscript substantially based on all reviewers' comments. The results are more meaningful and the discussion offers more depth and critical reflection now. I think the manuscript is now acceptable for publication, although I still think the quality and clarity of the writing could be improved in some instances, e.g. line 273 "Our systematic review shows that (...), while women in the USA and Australia did not." should be "while MIGRANT women in the USA and Australia did not".

Best regards

- **Response:**

**Thanks for your comment. We have edited this line 274 and have revised the whole manuscript to make it as clear as possible.**
